# CD4+ T cell help during early acute hepacivirus infection is critical for viral clearance and the generation of a liver-homing CD103+CD49a+ effector CD8+ T cell subset

**DOI:** 10.1371/journal.ppat.1012615

**Published:** 2024-10-11

**Authors:** Jarrett Lopez-Scarim, Dustyn Mendoza, Shashank M. Nambiar, Eva Billerbeck

**Affiliations:** 1 Division of Hepatology, Department of Medicine, Albert Einstein College of Medicine, Bronx, New York, United States of America; 2 Department of Microbiology and Immunology, Albert Einstein College of Medicine, Bronx, New York, United States of America; The University of North Carolina at Chapel Hill, UNITED STATES OF AMERICA

## Abstract

In hepatitis C virus (HCV) infection, CD4+ and CD8+ T cells are crucial for viral control. However, a detailed understanding of the kinetic of CD4+ T cell help and its role in the generation of different CD8+ T cell subsets during acute infection is lacking. The absence of a small HCV animal model has impeded mechanistic studies of hepatic antiviral T cell immunity and HCV vaccine development. In this study, we used a recently developed HCV-related rodent hepacivirus infection mouse model to investigate the impact of CD4+ T cell help on the hepatic CD8+ T cell response and viral clearance during hepacivirus infection in vivo. Our results revealed a specific kinetic of CD4+ T cell dependency during acute infection. Early CD4+ T cell help was essential for CD8+ T cell priming and viral clearance, while CD4+ T cells became dispensable during later stages of acute infection. Effector CD8+ T cells directly mediated timely hepacivirus clearance. An analysis of hepatic CD8+ T cells specific for two different viral epitopes revealed the induction of subsets of liver-homing CD103+CD49a+ and CD103-CD49a+ effector CD8+ T cells with elevated IFN-γ and TNF-α production. CD103+CD49a+ T cells further persisted as tissue-resident memory subsets. A lack of CD4+ T cell help and CD40L-CD40 interactions resulted in reduced effector functions and phenotypical changes in effector CD8+ T cells and a specific loss of the CD103+CD49a+ subset. In summary, our study shows that early CD4+ T cell help through CD40L signaling is essential for priming functional effector CD8+ T cell subsets, including unique liver-homing subsets, and hepacivirus clearance.

## Author summary

Chronic hepatitis C virus (HCV) infection affects about 50 million people worldwide and is a major cause for the development of end-stage liver disease. While chronic HCV infection can be cured, a prophylactic vaccine still does not exist. CD4+ and CD8+ T cells are important for HCV control, but a more in-depth understanding of the specific functions of T cell subsets during acute infection in the liver, the site of HCV infection, is needed for the development of effective vaccine strategies. Since access to human liver tissue is limited and HCV does not naturally infect mice, we previously developed a mouse model based on an HCV-related rodent hepacivirus. This model shares immunological features with human HCV infection and allows the study of antiviral immunity in the liver. In this study we used this model to assess the functional role of CD4+ and CD8+ T cell subsets during acute infection. We report a specific kinetic of CD4+ T cell dependent viral clearance and show that these cells provide critical help for the generation of unique liver-homing CD8+ T cell subsets. Overall, our study provides new insights into the determinates of hepacivirus clearance and immunity in the liver.

## Introduction

Acute hepatitis C virus (HCV) infection has two different outcomes: spontaneous clearance or development of a chronic infection [1]. Only about 30% of infected individuals can achieve acute clearance [2]. Chronic HCV infection affects approximately 50 million people worldwide and is a leading cause for the development of end-stage liver disease and hepatocellular carcinoma [2]. While chronic HCV infection can be cured with direct-acting antivirals (DAA) [1], a prophylactic vaccine that is likely needed for global HCV eradication does not exist [3].

Antiviral CD4+ and CD8+ T cells are crucial mediators of protective immunity during HCV infection and therefore represent important targets for HCV vaccine strategies [4–6]. However, an incomplete understanding of the generation of protective antiviral T cell responses in the liver impedes the development of successful T cell-based HCV vaccine strategies [6].

In general, during the early stages of a viral infection, virus-specific CD8+ T cells differentiate into various subsets that can broadly be divided by the expression of the IL-7 receptor α chain (CD127) and the inhibitor receptor KLRG-1 [7]. KLRG-1+CD127- short-lived effector cells expand rapidly during acute infection, exhibit antiviral effector functions, such as IFN-γ, TNF-α, granzyme B, and perforin production, and contract after viral clearance [7]. Heterogenous subsets of KLRG-1-/+CD127+ memory precursor cells develop into different types of memory cells after viral clearance, including tissue-resident memory (Trm) cells, which contribute to protection from secondary infection [7,8]. Expression of specific molecules, including the integrins CD49a and CD103 or the chemokine receptor CXCR6, mediate recruitment and retention of CD8+ T cell subsets to the infected tissue [8,9]. These molecules remain highly expressed on Trm cells after viral resolution [8,9].

CD4+ T cells provide essential help in priming the antiviral CD8+ T cell response [10,11]. Many studies using immunization and infection mouse models have shown that CD4+ T cell help is generally required to form functional memory CD8+ T cells [10,12]. The role of CD4+ T cell help in the induction of primary effector CD8+ T cells during acute infection is less clear and might differ between distinct viruses, viral tissue tropism and kinetics of the infection [10,11,13]. In addition, CD4+ T cells might also serve as effector cells that directly contribute to viral clearance.

During the acute phase of HCV infection, spontaneous clearance is associated with the presence of a robust and polyfunctional effector CD4+ and CD8+ T cell response in human patients [5,6]. In contrast, progression to viral persistence is linked to a lack of CD4+ T cells and CD8+ T cell dysfunction [5,6]. T cell depletion studies in chimpanzees showed that memory CD4+ T cell help is essential for CD8+ T cell mediated clearance of a secondary HCV infection [14,15].

HCV exclusively infects hepatocytes, and the liver microenvironment may play an important role in the generation of a functional antiviral T cell response [16]. Yet, there is limited knowledge of the induction of distinct hepatic effector and memory CD8+ T cell subsets, the contribution of specific effector functions to viral clearance, and the role of CD4+ T cells in providing help or acting as direct antiviral effectors during acute infection.

The study of hepatic immune mechanisms is impeded by limited access to human liver tissue and a lack of small immune-competent animal models for HCV [16,17]. However, we have previously developed a mouse model based on an HCV-related rodent hepacivirus (Norway rat hepacivirus [NrHV]), which allows for the mechanistic study of hepatic antiviral immunity [18]. NrHV infection in mice shares important similarities with human HCV infection such as exclusive hepatotropic infection, T cell dependent clearance of primary and secondary infection, and T cell mediated liver injury [18]. An infectious clone of mouse-adapted NrHV has been developed [19] and this model has been used to study antiviral hepatic immunity, including virus-specific CD8+ T cells, and genetic determinants of hepacivirus chronicity and pathogenesis [20,21]. Other variants of rodent hepacivirus have also been used to characterize antiviral T cells in mice [22].

In this study, we used the NrHV model to provide new insight into the direct contribution and kinetics of CD4+ and CD8+ T cells to acute hepacivirus clearance and into the characteristics of hepatic CD8+ T cells specific for distinct viral epitopes. We show that CD4+ T cell help, including CD40L-CD40 signaling, is required during early acute infection for the generation of functional effector CD8+ T cell subsets. CD8+ T cells directly mediate timely viral clearance. Interestingly, CD4+ T cells and CD40L-CD40 interactions were also specifically required for the induction of CD103+CD49a+ effector CD8+ T cell subsets. CD103+CD49a+ T cells were preferentially retained in the liver, showed increased INF-γ and TNF-α production and had the capacity to directly mediate viral clearance in vivo. They also persisted as long-term Trm cells in the liver indicating that these cells may play an important role in protective immunity to hepacivirus infection.

## Results

### CD4+ T cells are essential during early infection while CD8+ T cells are direct mediators of timely NrHV clearance

We have previously shown that infection with mouse-adapted NrHV results in robust hepatotropic infection, induction of antiviral T cell responses, and viral clearance at day 21–28 post-infection (pi) in C57BL/6 mice [18]. Mice receiving CD8+ T cell depletion antibodies showed delayed clearance suggesting an important role of these cells in virus elimination. We also showed that the absence of CD4+ T cells throughout acute NrHV infection (depletion prior to infection) leads to long-term viral chronicity, establishing an essential role for these cells in providing protective immunity [18]. Here, we set out to determine the impact of CD4+ T cells on the antiviral CD8+ T cell response and viral clearance.

**Fig 1:**
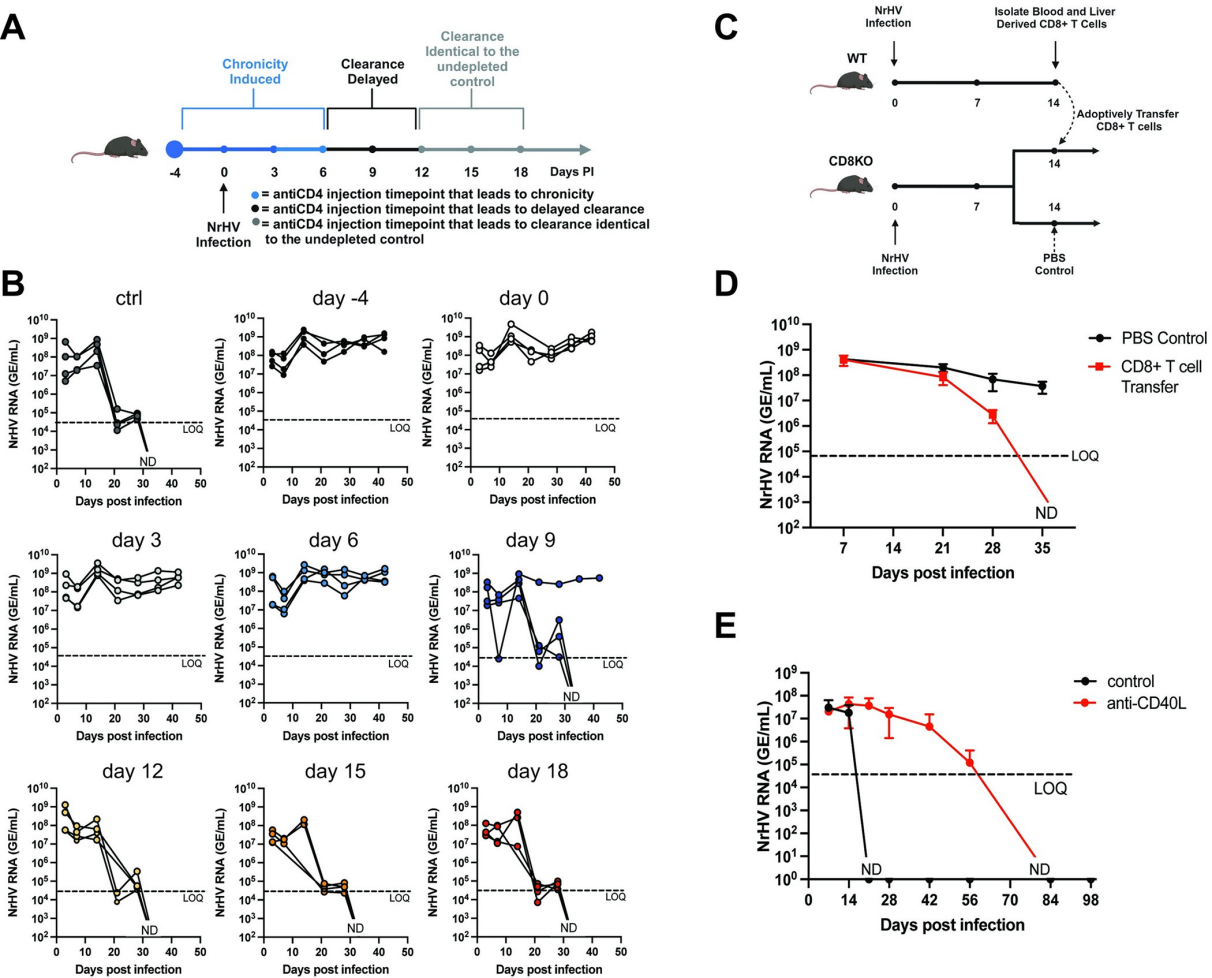
CD4+ T cell help during early NrHV infection is essential for viral clearance. (A-B) Groups of 8-week-old WT C57BL/6 mice (n = 4 per group) were infected intravenously with 10^4^ genome equivalents (GE) of NrHV and depleted of CD4+ T cells at specific time points using a depletion antibody. NrHV serum viral loads (NrHV RNA) were analyzed by RT-qPCR. (A) Outline of CD4+ T cell depletion time points and timeline of CD4+ T cell dependency for viral clearance. (B) NrHV viremia over time in groups of mice depleted of CD4+ T cells at indicated time points. (C-D) Adoptive transfer of WT-derived CD8+ T cells (6.9x10^4^ cells/mouse) into congenic CD8 knock-out (KO) mice. (C) Experimental outline. (D) NrHV viremia over time in experimental groups (n = 3–4). (E) Groups of mice (n = 6) were injected with an anti-CD40L antibody or isotype control at day 3 and 5 post NrHV infection. NrHV viremia over time is shown. Graphs show data for individual mice or mean with SD for groups of mice. LOQ: limit of quantification. ND: not detectable. Figure created with BioRender.com.

First, to define the kinetics of CD4+ T cell dependency during acute infection in more detail, we transiently depleted CD4+ T cells at selected time points post-infection in C57BL/6 mice (depletion start: day -4, 0, 3, 6, 7, 8, 9, 12, 15, 18 pi) (Fig 1A) and analyzed NrHV viremia over time. Interestingly, depletion at day 6 pi and earlier time points led to chronic infection while depletion starting at day 9 and later time points primarily resulted in viral clearance at day 21–28 pi similar to controls (Figs 1B and S1A: CD4 depletion control). Starting depletion at day 7 and 8 pi resulted in delayed clearance (day 42–77 pi, S1B Fig). These results reveal a specific kinetic of CD4+ T cell dependency during early infection. After day 9–12 pi, CD4+ T cells became dispensable for viral clearance despite high viral titers at these depletion time points (Fig 1A and 1B). It is conceivable that an essential role of CD4+ T cells during early NrHV infection is to provide help for priming of effector CD8+ T cells which then mediate viral clearance. However, CD4+ T cells can also provide help to B cells. In fact, it has recently been shown that both, virus-specific antibodies and CD8+ T cells are important for NrHV clearance [23].

To directly show that CD8+ T cells are essential mediators of timely viral clearance, we adoptively transferred NrHV-primed CD8+ T cells (isolated at day 14 pi from WT C57BL/6 mice) into congenic CD8^-/-^ mice at day 14 pi (Fig 1C). These mice have functional CD4+ T cells and B cells and only lack CD8+ T cells. CD8^-/-^ mice showed prolonged but variable duration of infection which could last over 90 days pi, but clear as early as day 42 pi in some mice (S1D Fig). However, adoptive transfer of T cells consistently resulted in viral clearance at earlier time points as compared to the majority of mice in PBS-control CD8^-/-^ groups. (Figs 1D and S1D). While it is not possible to confirm the successful transfer and homing of CD8+ T cells to the liver of CD8^-/-^ mice during this experiment, we analyzed the livers of these groups of mice several months after infection. CD8+ T cells were present at varying degrees in the livers of mice from the adoptive transfer group and completely absent in the PBS control group (S1C Fig), showing that CD8+ T cells were efficiently transferred and maintained long-term in CD8^-/-^ mice.

Thus, CD8+ T cells directly mediate timely viral clearance during acute NrHV infection. A lack of CD4+ T cells likely impacts the efficacy of these cells contributing to delayed clearance or chronic infection (Fig 1A).

One mechanism of CD4+ help is the CD40L-CD40 mediated licensing of dendritic cell (DC) subsets for antigen cross-presentation to CD8+ T cells [10]. Antibody-mediated blockade of this interaction during early NrHV infection led to prolonged viremia (clearance day 42-84pi) in treated mice as compared to controls (Fig 1E), indicating that CD40L-CD40 interactions plays an important role in hepacivirus clearance.

Overall, our data suggest that early CD4+ T cell help is essential for priming protective effector CD8+ T cell responses during acute hepacivirus infection. However, it is possible that CD4+ T cell depletion and blockade of CD40L-CD40 signaling impacts humoral immunity, which is also essential for NrHV clearance [23]. In fact, we found that global IgG production was significantly reduced in CD40L blocked mice at day 35 pi as compared to control mice (S1E Fig). In summary, prolonged infection and chronicity as shown in Fig 1B and 1E are likely due to an impairment of both, the T cell and the B cell response. However, this study is focused only on the CD8+ T cell response.

### Analysis of hepatic CD8+ T cells specific for two different NrHV epitopes

Next, we aimed to determine the impact of CD4+ T cells and CD40L-CD40 interactions on distinct NrHV-specific CD8+ T cell subsets and their functionality. However, the NrHV mouse model is relatively new and virus-specific CD8+ T cell subsets, their kinetics and their effector functions are not yet well characterized in this model. Thus, we first performed a high-dimensional flow cytometric analysis of hepatic CD8+ T cell subsets targeting two different NrHV epitopes during acute infection (day 7, 14, 21, 28 pi), post clearance (day 42 pi) and memory stage (day 150 pi). We analyzed CD8+ T cells using MHC class I tetramers specific for two immune-dominant NrHV epitopes located in NS3 and NS4 proteins as described previously [20]. We focused our analysis on the liver, the exclusive site of infection, and the major site of T cell activation during NrHV infection [18].

**Fig 2:**
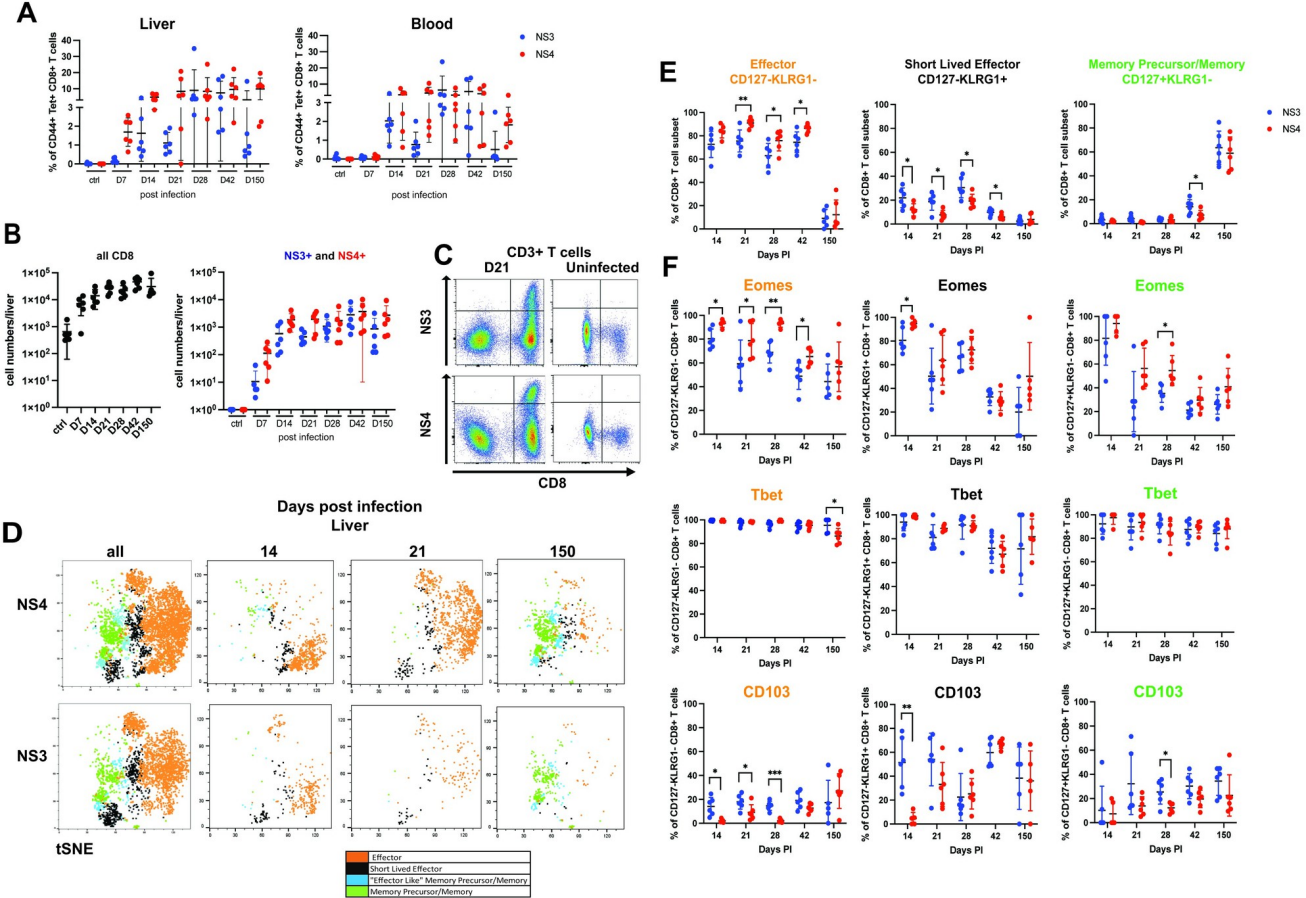
Hepatic CD8+ T cell responses to different viral epitopes during NrHV infection. Groups of WT mice (n = 6) were infected with 10^4^ GE NrHV. At indicated time points post infection (pi) and in uninfected controls hepatic and peripheral lymphocytes were analyzed for CD8+ T cell responses specific for a NrHV-NS3 and a NrHV-NS4 epitope using MHC class I tetramers and spectral flow cytometry. (A) Frequencies of NS3- and NS4-specific CD8+ T cells in liver and blood. (B) Total hepatic cell numbers. (C) Representative FACS plot of NS3 and NS4 MHC class I tetramer staining for hepatic CD3+ T cells. (D) tSNE representation of flow data showing the kinetics of the following NS3- and NS4-specific subsets: effector (CD127-KLRG-1-); short lived effector (CD127-KLRG-1+); effector like memory precursor (day 7–42 pi) or memory (day 150 pi) (CD127+KLRG-1+); and memory precursor (day 7–42 pi) or memory (day 150pi) (CD127+KLRG-1-). (E) Frequencies of the different NS3- and NS4-specific subsets in the liver at indicated time points. (F) Eomes, T-bet and CD103 expression levels in the different virus-specific CD8+ T cell subset. Graphs show mean with SD. Statistics: one- or two-way ANOVA with Tukey multiple comparison test; * p<0.05, ** p<0.01, *** p<0.001, **** p<0.0001.

Hepatic NS3- and NS4-specific CD8+ T cells became readily detectable at day 14 pi and reached peak frequencies and numbers at day 21–28 pi without a significant contraction weeks after clearance (day 42 pi). NS3+ and NS4+ cells persisted at least until day 150 pi in the liver, (Figs 2A–2C and S2). Virus-specific cells were also detectable in peripheral blood. Overall, we observed significant variability in tetramer+ cells between individual mice throughout our study, with NS3+ and NS4+ cells ranging from 1–20% in frequency of all hepatic CD8+ T cells and 10^2^−10^4^ in total numbers.

Using t-distributed stochastic neighbor embedding (t-SNE), we visualized changes in CD8+ T cell subsets over time (Fig 2D). During acute infection, hepatic NS3+ and NS4+ cells showed a CD44+CD127-KLRG-1-/+ effector phenotype. Interestingly, only 10–20% of effector cells expressed KLRG-1, a classical marker of short-lived effector cells (SLEC) (Figs 2D, 2E and S2: gating strategy). The KLRG-1- effector subset persisted until 2 weeks after clearance (day 42 pi). At day 150 pi, the majority of cells showed a KLRG-1-CD127+ memory phenotype with expression of the memory-associated transcription factor TCF-1 (Figs 2D, 2E and S3A).

The majority of effector cells expressed the transcription factors T-bet and Eomes, which mediate CD8+ T cell effector functions (Fig 2F) but did not express TCF-1 (S3A Fig). We also observed proliferation (Ki67 expression) and upregulation of the cytotoxicity associated factor CX3CR1, at day 14 pi (S3B and S3C Fig).

We detected distinct phenotypical differences between effector NS3+ and NS4+ CD8+ T cells. A higher percentage of NS4+ cells expressed Eomes and CX3CR1 as compared to NS3+ cells (Figs 2F and S3B). Interestingly, NrHV infection induced subsets of CD103+ effector and memory precursor/memory cells, which were enriched in the NS3+ population (Fig 2F). CD103, an integrin that mediates tissue retention, is highly expressed by Trm cells in certain tissues [8]. Although, Trm cells in the mouse liver do not usually express CD103 [24]. Further, a role for CD103+ effector cells during acute hepatic viral infection has not been described so far.

Overall, our analysis provides a detailed kinetic and characterization of the CD8+ T cell response specific for two viral epitopes. Our results align with previous reports of the hepatic antiviral CD8+ T cell response during NrHV infection [18,22]. In addition, we identified epitope-specific differences and an unusual virus-specific CD103+ subset.

### Effector functions of NrHV-specific CD8+ T cells

**Fig 3:**
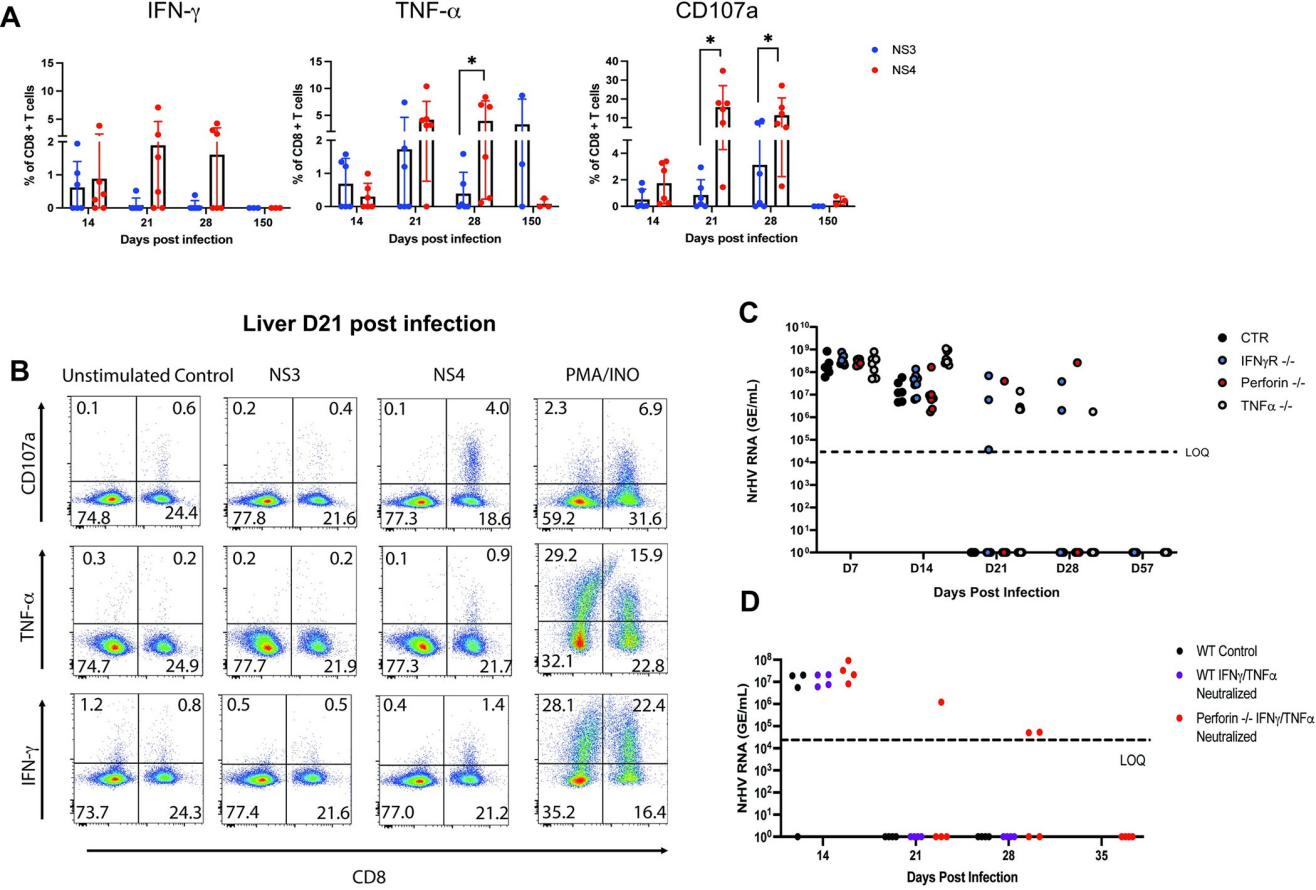
CD8+ T cell effector functions in NrHV clearance. (A) Hepatic CD8+ T cells were isolated from NrHV-infected WT mice (n = 6) at indicated points. IFN-γ, TNF-α and CD107a expression in response to 5h in vitro stimulation with the NS3 or NS4 peptide was assessed by flow cytometry. Graphs show frequencies of IFN-γ+, TNF-α+ or CD107a+ CD8+ T cells after subtraction from negative control (unstimulated cells). (B) Representative FACS plots of data shown in panel A. (C) NrHV viremia over time in IFNγR^-/-^, TNFα^-/-^, Perforin^-/-^ and WT control mice (n = 4–8). (D) NrHV viremia in control WT mice and Perforin^-/-^ mice with or without administration of neutralizing antibodies for IFN-γ and TNF-α at 12,15,18,21 post NrHV infection (n = 4 per group). Graphs show mean with SD or only data for individual mice (C). LOQ: limit of quantification. Statistics: unpaired two-tailed t test: * p<0.05, ** p<0.01, *** p<0.001, **** p<0.0001.

Next, we analyzed the functional properties of NS3+ and NS4+ cells and assessed the direct role of major CD8+ T cell effector functions in NrHV clearance. CD8+ T cells were polyfunctional as indicated by IFN-γ, TNF-α, and CD107a (cytolytic degranulation) production in response to NS3 and NS4 peptide stimulation during acute infection (Figs 3A, 3B and S4A). Consistent with the high variability of NS4+ and NS3+ cell numbers in individual mice (Fig 2A and 2B), we observed variability in effector functions with peptide stimulation. Interestingly, NS4 peptide stimulation resulted in significantly higher CD107a induction as compared to NS3 peptide stimulation at two time points (Fig 3A). We performed tetramer/cytokine co-staining to directly show that NS4-specific cells produced CD107a to a higher extent than IFN-γ or TNF-α (S4B–S4D Fig). These results are in line with a recent study showing strong CD107a expression by CD8+ T cells specific for a different rodent hepacivirus variant [22].

To assess if cytokine-mediated or cytolytic effector functions play a dominant role in NrHV clearance, we analyzed the course of infection in TNF-α^-/-^, IFNγR^-/-^ and Perforin^-/-^ mice. Most mice from the 3 knock-out strains cleared the infection at day 21–28 pi similar to control mice, while some individual mice from all knock-out strains showed delayed clearance (day 35–56 pi) (Fig 3C). Antibody-mediated neutralization of both IFN-γ and TNF-α throughout acute infection in WT control mice or Perforin^-/-^ mice resulted in timely clearance (day 21 pi) in control mice and delayed clearance (day 35 pi) in 50% of Perforin^-/-^ mice (Fig 3D). Of note, viral clearance in Perforin-/- mice with or without IFN-γ and TNF-α neutralization could be mediated by compensatory mechanisms such as Fas-FasL mediated cytotoxicity as has been shown for a different infection model [25].

In summary, these results show that the lack of specific effector functions can result in delayed clearance, however there is no dominant role of any effector function in NrHV clearance.

Overall, our phenotypical (Fig 2) and functional (Fig 3) analyses of the antiviral CD8+ T cell response during NrHV infection show that viral clearance is associated with the emergence of polyfunctional CD8+ T cells specific for at least two different immune-dominant epitopes. Our in vivo data supports studies from HCV patients which established an association of spontaneous clearance with the emergence of polyfunctional HCV-specific CD8+ T cells and suggested a role for both, cytokine-mediated and cytolytic effector functions, in viral clearance [5,6,26].

### A lack of CD4+ T cell help leads to multilayered impairment of effector CD8+ T cells during acute infection

**Fig 4:**
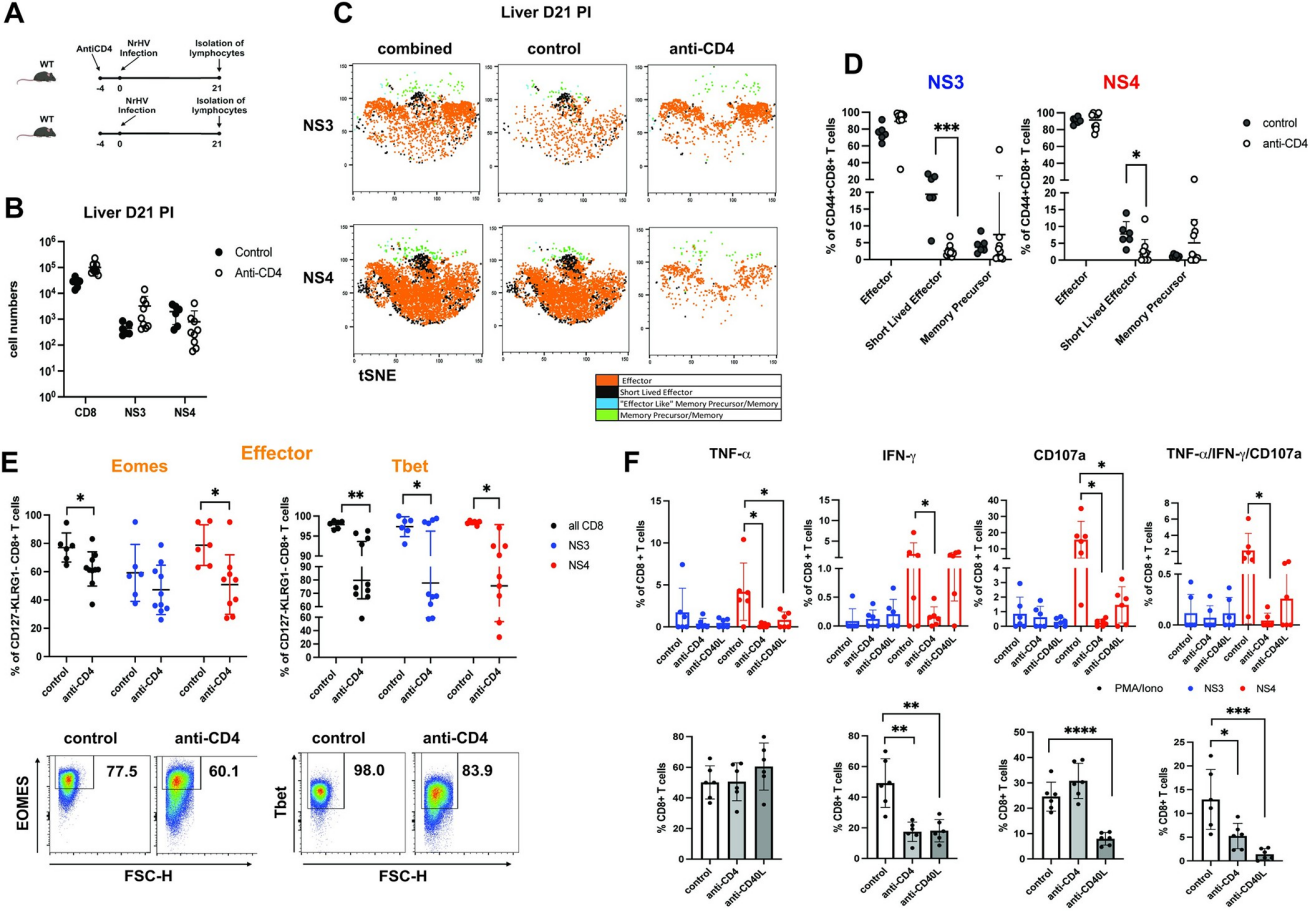
Phenotypical and functional changes in hepatic effector CD8+ T cell subsets lacking CD4+ T cell help. WT mice (n = 6–9 per group) were either depleted of CD4+ T cells 4 days prior to NrHV infection (anti-CD4) or not depleted (control). At day 21 pi hepatic and peripheral CD8+ T cells were analyzed using spectral flow cytometry. (A) Experimental outline. (B) Total hepatic cell numbers of all CD8+ T cells, NS3- and NS4-specific cells. (C) tSNE representation of flow data showing hepatic NS3- and NS4-specific CD8+ T cell subsets (exactly as described in Fig 2) in control and CD4-depleted mice. (D) Frequencies of NS3- and NS4- specific subsets in both groups of mice. (E) Upper panel: Eomes and T-bet expression in the general (all CD8), NS3- and NS4-specific effector subsets. Lower panel: Representative FACS plots (of concatenated samples from each group). (F) Groups of control mice, CD4+ depleted mice or CD40L blocked mice (CD40L blocking antibody: day 3 and 5 pi) were analyzed for CD8 effector functions at day 21 pi (n = 6 per group). Frequencies (subtracted from negative unstimulated control) of TNF-α, IFN-γ, CD107a and polyfunctional (IFN-γ+TNF-α+CD107a+) CD8+ T cell responses after 5h in vitro stimulation with the NS3 or NS4 peptide (upper panel) or unspecific stimulation with PMA/Ionomycin (lower panel). Graphs show mean with SD. Statistics: unpaired two-tailed t test; * p<0.05, ** p<0.01, *** p<0.001, **** p<0.0001. Figure created with BioRender.com.

Based on these analyses we then determined the impact of CD4+ T cell help on virus-specific CD8+ T cell subsets and their functionality. Since both, hepatic NS3+ and NS4+ cells were detectable at sufficient numbers at day 21 pi (time point of clearance) (Fig 2B), we chose this time point to compare CD8+ T cells from mice depleted of CD4+ T cells 4 days prior to infection and non-depleted controls (Fig 4A). A lack of CD4+ T cells resulted in slightly reduced total hepatic numbers of NS4+ but not NS3+ cells indicating that virus-specific CD8+ T cells can be generated without CD4+ T cell help albeit at lower numbers (Fig 4B). t-SNE analysis showed significant phenotypical changes in NS3+ and NS4+ cells generated with or without help (Fig 4C). Overall, there was a significant loss of the SLEC subset within the unhelped NS3+ and NS4+ populations (Fig 4C and 4D). Global CD8+, NS3+ and NS4+ effector and memory precursor T cell subsets showed reduced expression of T-bet and Eomes in the absence of CD4+ T cells (Figs 4E and S5A). Unhelped NS3+ effector cells further showed significant upregulation of the inhibitory receptors LAG-3 and Tim-3. Interestingly, Ly108 (SLAMF6) which is a marker of progenitor exhausted CD8+ T cells during chronic viral infections [27], was also upregulated in unhelped NS3+ and NS4+ cells at day 21 pi (S5B and S6B Figs). CD107a, IFN-γ, TNF-α expression and polyfunctionality (TNFα+IFN-γ+CD107a+) were also significantly impaired in global and/or NS4+ CD8+ T cells without CD4+ help (Figs 4F and S6A).

In summary, the combined loss of SLEC, downregulation of T-bet and Eomes, upregulation of inhibitory receptors and polyfunctional impairment within the hepatic CD8+ T cell population during the acute phase is likely a major factor in the establishment of chronic NrHV infection in mice lacking CD4+ T cell help (Fig 1B). To assess if CD4+ T cell help to NrHV-specific CD8+ T cells is mediated through CD40L-CD40 interactions we analyzed the CD8+ T cell response at day 21 pi in mice that received CD40L blocking antibody at day 3 and 5 pi. In these mice hepatic NS3+ and NS4+ cell developed at numbers comparable to control and CD4+ depleted mice (S5C Fig). There were no consistent significant changes in T-bet, Eomes or inhibitory receptor expression with CD40L blockade (S5D–S5E Fig). However, like a lack of CD4+ T cells, the interruption of CD40L-CD40 signaling resulted in significantly reduced CD8+ T cell effector functions after peptide-specific or PMA/Ionomycin stimulation (Figs 4F and S6A). These results show that CD4+ T cell mediated CD40L-CD40 interactions have a direct impact on the functionality of the antiviral CD8+ T cell response which likely contributes to the delayed viral clearance in mice treated with CD40L blocking antibody (Fig 1E).

Together, our in vivo depletion study directly shows that early CD4+ T cell help, by mechanisms including CD40L-CD40 signaling, is essential for the generation of functional anti-hepaciviral effector CD8+ T cells. This is in line with studies from acute HCV infection in humans where undetectable CD4+ T cells and effector CD8+ T cells with limited effector functions have been correlated with progression to chronic infection [5,26].

### CD4+ T cell help promotes the generation of a CD103+CD49a+ effector CD8+ T cell subset

**Fig 5:**
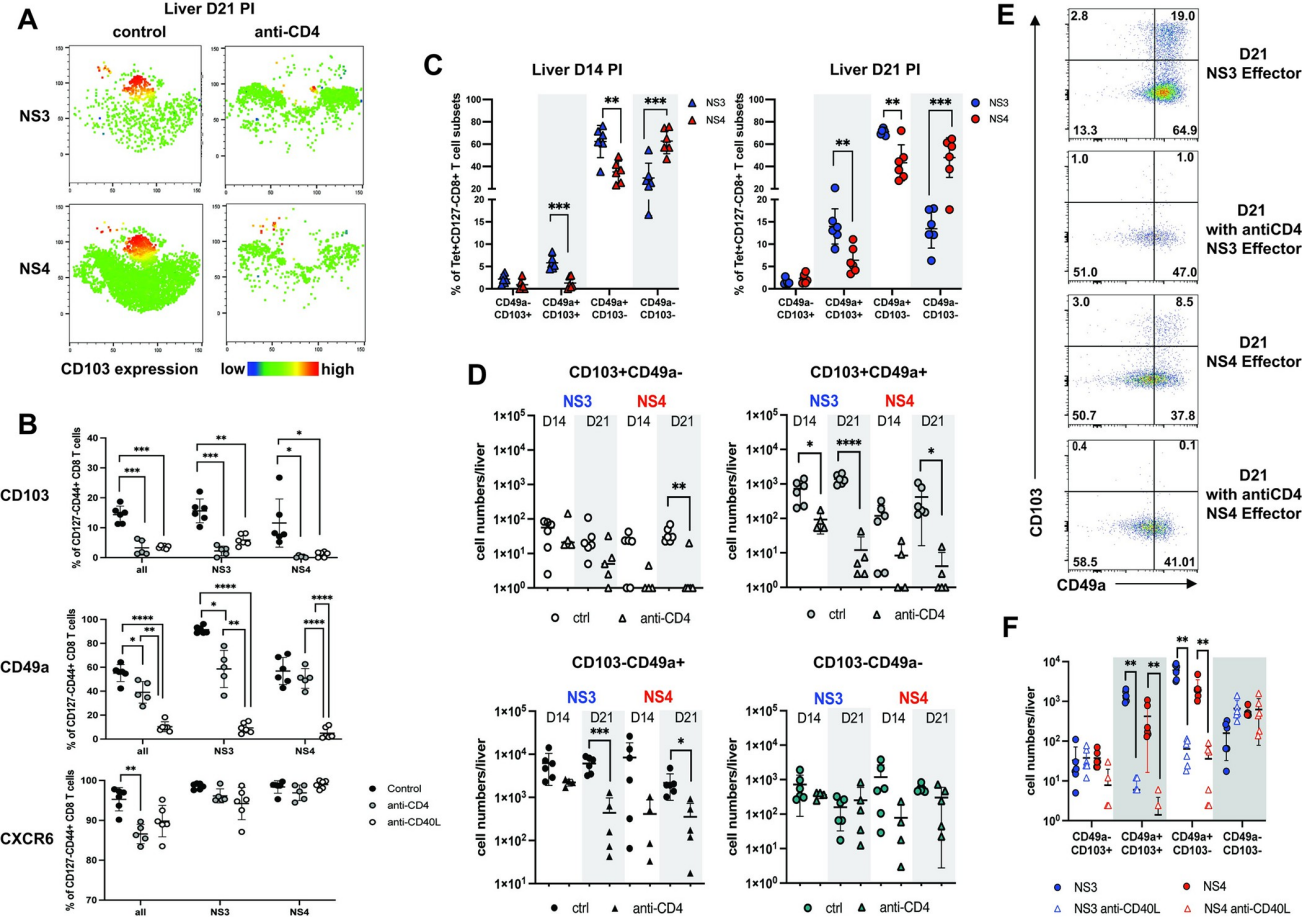
CD4+ T cell help promotes the induction of a CD103+CD49a+ effector CD8+ T cell subset. (A) CD103 expression levels for the tSNE plots shown in Fig 4C(B) CD103+, CD49a+ and CXCR6+ cells within the hepatic general, NS3- and NS4-specific effector CD8+ T cell subsets at day 21 pi in control, CD4-depleted and CD40L blocked mice (CD40L blocking antibody: day 3 and 5 pi) (n = 6 per group). (C-E) WT mice (n = 4–6) were depleted of CD4+ T cells 4 days prior to NrHV infection (anti-CD4) or not depleted (control). At day 14 and 21 pi hepatic and peripheral NS3- and NS4-specific CD8+ T cells were analyzed for CD103 and CD49a co-expression using spectral flow cytometry. (C) Frequencies of CD103+ and/or CD49a+ cells in NS3- and NS4-specific effector cells (CD127-KLRG-1+/-) of control mice. (D) Total hepatic cell numbers of CD103/CD49a subsets at day 14 and 21 pi in control and CD4-depleted mice. (E) Original FACS plots showing CD103 and CD49a expression on NS3- and NS4-specific cells with or without CD4 T cell help. (F) Control mice and CD40L blocked mice were analyzed for NS3+ and NS4+ CD103/CD49a subsets at day 21 pi. Total hepatic effector cell numbers are shown. Graphs show mean with SD. Statistics: unpaired two-tailed t test or one-way ANOVA with Tukey multiple comparison test; * p< 0.05, ** p< 0.01, *** p<0.001, **** p<0.0001.

Interestingly, a lack of CD4+ T cells also resulted in the specific reduction of CD103+ effector CD8+ T cell subsets at day 21 pi (Figs 5A and S5F). This was particularly pronounced in the NS3+ cell population, which contained a higher percentage of CD103 expressing cells (Fig 2).

To investigate this further and to test if CD40L blockade has a similar effect, we next analyzed all hepatic effector cells (CD127-KLRG-1+/-) from CD4+ depleted groups and groups receiving CD40L blocking antibody for expression of CD103 and other markers of tissue-retention, specifically CD49a and CXCR6 (Figs 5B and S2). CD69 expression, another important marker of tissue-residency [28] was not analyzed during acute infection, since it is also an activation marker which is highly expressed by activated T cells. A lack of CD4+ T cells and CD40L signaling resulted in significantly reduced frequencies of global, NS3+ and NS4+ CD103 and CD49a expressing cells, while CXCR6 expression was not as strongly affected (Fig 5B).

Of note, at day 21 pi CD4+ depleted mice show high titer viremia, while non-depleted mice have mostly cleared the infection (Fig 1B). To investigate whether the reduced frequencies of CD103+ and CD49a+ cells is due to differences in viral load or linked to a lack of CD4+ help, we next analyzed CD4+ depleted and non-depleted mice at day 14 (when both groups show high titer viremia) and at day 21 pi. We found reduced but detectable total numbers of NS3+ and NS4+ CD8+ T cells at both time points in the liver of depleted mice (S7A Fig). We then assessed CD103 and CD49a co-expression on all effector cells. In non-depleted mice, a large proportion of hepatic CD103+CD8+ T cells co-expressed CD49a (Fig 5C–5D). We observed significantly higher frequencies of CD103+CD49a+ and CD103-CD49a+ cells within the NS3+ population. The NS4+ population in contrast showed higher frequencies of CD103-CD49a- cells (Fig 5C). CD103+CD49a+ cells increased between day 14 to day 21 pi. These differences in frequencies were consistent with total hepatic cell numbers of the respective subsets (Fig 5D).

A lack of CD4+ T cell help resulted in a significant decrease of total cell numbers and frequencies of CD103 and/or CD49a expressing NS3+ and NS4+ cells at day 14 and/or day 21 pi. This effect was most significant for the CD103+CD49a+ subset. Numbers of CD103-CD49a- cells were not significantly altered by the lack of CD4+ T cell help (Figs 5D–5E and S7B). CD40L blockade resulted in a similar reduction of CD103+ and/or CD49a+ cell numbers at day 21 pi (Fig 5F**).**

Together, this data shows that NrHV infection induces subsets of CD103+CD49a+ virus-specific effector CD8+ T cells that are reduced in the absence of CD4+ T cell help and CD40L-CD40 interactions.

### Liver-homing and functional properties of CD103+CD49a+ effector CD8+ T cells

Since CD103+CD49a+ effector CD8+ T cells were significantly impacted by a lack of CD4+ T cell help and CD40L signaling we wanted to characterize this unusual subset in more detail. First, we assessed whether CD103 and/or CD49a expression is associated with liver retention of CD8+ T cells during acute infection. CD49a expression was strongly linked to liver homing/retention as frequencies of CD49a+ NS3+ and NS4+ cells were significantly higher in the liver compared to peripheral blood. In contrast, CD103 expression by itself was not associated with hepatic cells (S7C Fig).

**Fig 6:**
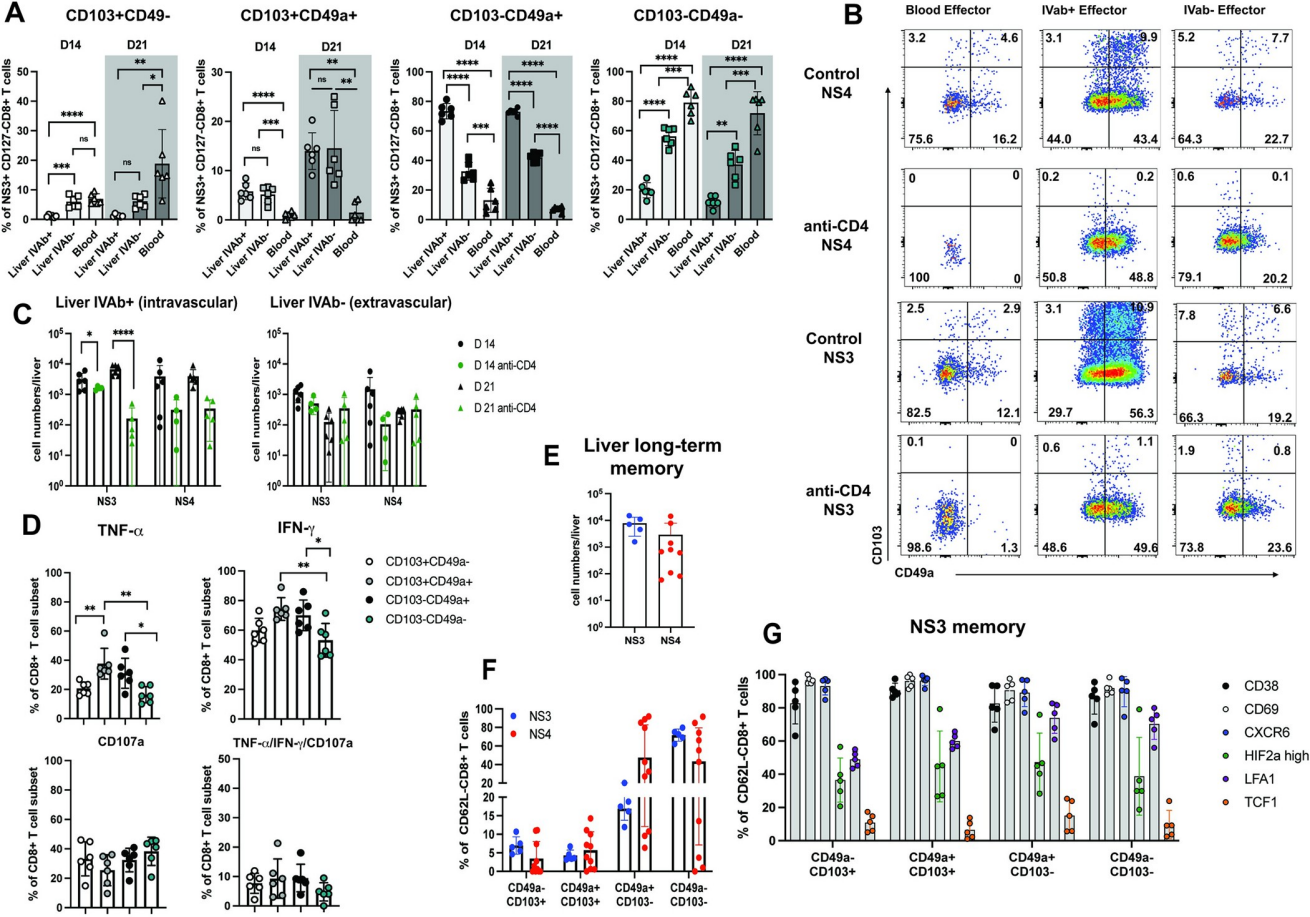
Liver-homing and functional properties of CD103+CD49a+ effector CD8+ T cells. (A) CD8 intravascular staining was performed to distinguish intravascular (IVAb+) from extravascular (IVAb-) T cells. Graphs show percentages of CD103/CD49a subsets within the NS3-specific population from liver IVAb+, liver IVAb- and peripheral blood at day 14 and 21 pi in WT control mice (n = 6). (B) Original FACS plots showing CD103/CD49a subsets within the NS4- and NS3-specific populations at day 21 pi. (C) Total cell numbers of NS3- and NS4-specific cells in the liver IVAb+, liver IVAb- compartment at day 14 and 21 pi (n = 6). (D) TNF-α, IFN-γ and/or CD107a expression of hepatic CD103/CD49a CD8+ T cell subsets after unspecific stimulation with PMA/Ionomycin at day 21 pi in control mice. (E) Total numbers of hepatic NS3- and NS4-specific CD62L- memory T cells at day 220 pi. (F) Percentages of CD103/CD49a subsets within the NS3- and NS4-specific memory population at day 220 pi. (G) CD38, CD69, CXCR6, LFA-1, HIF-2a and TCF-1 expression in the respective NS3+ memory CD103/CD49a subsets. Statistics: one or two-way ANOVA with Tukey multiple comparison test or unpaired t test; * p<0.05, ** p<0.01, *** p<0.001, **** p<0.0001.

It has been shown that liver resident CD8+ T cells can remain in the liver vasculature (sinusoids) or localize into the tissue [29–31]. To determine the location of CD103/CD49a subsets in the liver and to assess the impact of CD4+ T cell help on it, we performed CD8 intravascular staining [32]. This allows to distinguish intravascular (IVAb+) from tissue-localized (IVAb-) CD8+ T cells. We found distinct localization patterns of CD8+ T cells based on CD49a and CD103 expression (Fig 6A–6B). For example, CD103-CD49a+ cells were enriched in the IVAb+ fraction. CD103+CD49a+ cells, the subset most affected by CD4 depletion, were detected at similar frequencies in the IVAb+ and IVAb- fraction (Fig 6A–6B). These cells were significantly reduced in all compartments in mice depleted of CD4+ T cells (Figs 6B and S7D). We observed the most significant overall reduction of virus-specific CD8+ T cells in the absence of help in the IVAb+ NS3+ CD8+ T cell compartment (Figs 6C and S8A). Together, these results show that NrHV-specific CD103+CD49a+ effector cells are specifically retained in the liver vasculature and tissue during NrHV infection and that a lack of CD4+ T cell help leads to a general loss of these cells.

CD103 and CD49a expression are induced by TGF-β [8,9]. Interestingly, NrHV infection was associated with increased serum TGF-β levels at day 14 pi in control mice but not CD4+ depleted mice (S8B Fig). TGF-β levels in liver-tissue did not increase during infection and were not affected by a lack of CD4+ T cells (S8C Fig). In addition, we observed no statistically significant differences in the numbers of hepatic TGF-β+ CD4+ T cells at day 14 pi and uninfected control mice (S8D Fig). However, TGF-β can be produced by many cell types and differences in serum versus tissue levels at day 14 pi could be due to the kinetics and location of expression during infection. Interestingly, IL-15 was significantly reduced in the liver of CD4+ depleted mice (S8C Fig). IL-15 acts together with TGF-β to promote the generation and maintenance of tissue-resident T cells [33].

Overall, our data suggest that systemic TGF-β and local IL-15 secretion may contribute to CD103 and CD49a co-expression and liver retention of virus-specific CD8+ T cells during acute NrHV infection.

We next analyzed if CD103 and CD49a expression were associated with functional differences in effector CD8+ T cells. Given the high variability in response to NS3 and NS4 peptide stimulation (Figs 3 and 4) and the low overall numbers of some CD103/CD49a subsets, we analyzed global hepatic CD8+ T cells after unspecific PMA/Ionomycin stimulation isolated at day 21 pi. Interestingly, CD103+CD49a+ and CD103-CD49a+ subsets displayed significantly higher TNF-α and IFN-γ expression as compared to CD49a- subsets. CD107a levels were slightly, but not statistically significant, reduced in CD103+CD49a+ cells (Fig 6D). To directly assess if CD103/CD49a subsets have the capacity to clear NrHV infection, we adoptively transferred sorted CD103+CD49+, CD103-CD49a+ and CD103-CD49a- effector cells into CD8^-/-^ mice (Experimental outline similar to Fig 1D). For this experiment, given the lower number of CD103+CD49a+ cells, we were able to only transfer 6x10^3^ cells of each subset into individual recipient mice as compared to 6.9x10^4^ cells/mouse for a general CD8+ T cell transfer. However, despite the lower number of transferred cells, we found that some mice receiving CD103+CD49a+ or CD103-CD49a+ cells, achieved viral clearance at similar time points as mice that received all CD8+ T cells. None of the PBS control group achieved NrHV clearance at these time points (S7E Fig). These results suggest that CD103-/+CD49a+ cells can represent efficient effector CD8+ T cell subsets in vivo.

Finally, we determined if CD103+CD49a+ cells have the potential to persist as long-term Trm cells in the liver after viral resolution. We analyzed groups of mice at day 220 pi for CD103, CD49a, and other markers associated with liver Trm cells such as LFA-1, CD69, CXCR6, HIF2a and CD38 [28,29,34,35]. It has to be noted that memory T cell formation cannot be analyzed in mice depleted of CD4+ T cells prior to infection as these mice develop long-term chronic infection. Hepatic NS3+ and NS4+ CD62L-CD8+ T cells were readily detectable at day 220pi (Fig 6E). The majority of NS3+ and NS4+ memory cells were CD103-CD49a-/+, but about 5–15% co-expressed CD103 and CD49a (Fig 6F), which is similar to the frequency of these cells during acute infection (Fig 5C). 80–100% of CD103+/-CD49a+ cells co-expressed CD69, CD38 and CXCR6 while TCF-1 expression was reduced (Figs 6G and S8E). LFA-1 expression was significantly higher in cells negative for CD103 (S8F Fig). HIF-2a was expressed at similar levels in all subsets (S8F Fig). Overall, this data shows that the CD103+CD49a+ subset represents a Trm cell subset with expression of the classical tissue-retention markers CD69, CD38 and CXCR6 [8,28].

Upon secondary infection, CD103+CD49a+ and CD103-CD49a+ cells rapidly expanded (S9A–S9D Fig), and produced cytokines (S9E Fig), indicating that both subsets contribute to the recall response. We previously showed that secondary infection is controlled by CD8+ T cells [18].

In summary, our data shows that a hepatic virus-specific CD103+CD49a+ subset, which is significantly impacted by a lack of CD4+ T cell help and CD40L signaling, is not only present during the effector response but also persists as a long-term Trm population.

## Discussion

In this study, we used an HCV-related rodent hepacivirus mouse model to analyze the impact of CD4+ T cell help on viral clearance and the generation of different virus-specific CD8+ T cell subsets. While CD4+ and CD8+ T cells are already known to play an important role in HCV infection, the lack of an immune-competent HCV mouse model has made it difficult to study the role of hepatic T cell subsets during acute infection [5,6,16]. The NrHV model may aid in HCV vaccine development by allowing the study of hepatic antiviral immune responses during an exclusive hepatotropic virus infection in vivo.

By performing a CD4+ T cell depletion kinetic, we showed that the presence of CD4+ T cells during early acute infection is required for viral clearance. In contrast, CD4+ T cells are not required for viral clearance at later stages of infection. With adoptive transfer experiments, we further showed that timely virus elimination is directly mediated by CD8+ T cells, which is consistent with our previous CD8+ T cell depletion experiments [18]. Together, our results indicate that a main function of CD4+ T cells during acute hepacivirus infection is to provide help in T cell priming and that, unlike in some other viral infections [10], the primary anti-hepaciviral effector T cell response is help dependent. This finding is in line with the observation that spontaneous HCV clearance in human patients is associated with the presence of a strong CD4+ T cell response [5,26].

It is important to note that the use of a CD4+ T cell depletion antibody can cause potential off-target effects by depleting other cell subsets expressing CD4 [36].

However, we used an additional approach to target CD4+ T cell help. A major mechanism of CD4+ T cell help is the CD40L-CD40 mediated licensing of conventional DCs for priming of CD8+ T cells [10]. Our finding that CD40L blockade during early infection leads to prolonged viremia further supports that CD8+ T cell priming is an essential function of CD4+ T cells and indicates that the CD40L-CD40 pathway plays an important role in hepacivirus infection.

A recent study showed that in addition to CD8+ T cells, virus-specific IgG is essential for rodent hepacivirus clearance [23]. Therefore, a lack of CD4+ T cell help or CD40L signaling during early infection might also impact the B cell response. This is supported by our finding of reduced IgG levels in the serum of CD40L blocked mice at day 35 pi. Clearly, prolonged infection and chronicity in CD4+ depleted and CD40L blocked mice are likely due to an impairment of both, the T cell and the B cell response. The role of CD4+ T cell help for humoral immunity in this model should be addressed in future studies. Our study was exclusively focused on the impact of CD4+ T cells on the CD8+ T cell response.

The virus-specific hepatic CD8+ T cell response is not well described yet in the relatively new NrHV mouse model. Therefore, we initiated our study with a detailed characterization of the kinetics, phenotype and function of CD8+ T cell responses specific for two immune-dominant NrHV epitopes that we previously described [20].

Our analysis of NS3- and NS4-specific CD8+ T cell responses during acute infection and memory stage showed that NrHV infection induces a strong hepatic antiviral effector CD8+ T cell response and that memory CD8+ T cells persist in the liver. This finding is consistent with our previous study and another recent report [18,22]. Interestingly, we did not observe a significant contraction of the effector response after viral clearance (day 21-35pi). It is, therefore, possible that small amounts of virus below the limit of detection of our qPCR assay persist for longer in the liver. However, at day 150 pi virus-specific cells showed a memory phenotype. It will be interesting to analyze additional time points between day 42 and 150 pi to further investigate the transition from effector to memory subsets. In addition, a major marker of short-lived effector cells, KLRG-1, was only expressed on 10–20% of effector cells during acute infection. KLRG-1 expression is dynamic [37], and it is possible that there is a fast turnover of these cells in the liver. KLRG-1 is also downregulated by TGF-β, which we found to be increased in the serum during NrHV infection [38]. Our analysis also revealed epitope-specific difference in effector subsets highlighting the importance of assessing a broader range of the antiviral CD8+ T cell response. We found that a higher percentage of NS4-specific cell exhibit a cytolytic effector phenotype with elevated Eomes and CD107a expression, while the NS3-specific subset contained more cells with a CD103+CD49a+ tissue resident phenotype. The origin and functional consequences of these findings need to be further investigated.

Interestingly, epitope-specific difference in CD8+ T cell subsets have been reported in other viral infections. In an influenza mouse model, lung memory CD8+ T cells specific for two different immune-dominant epitopes in influenza NP (nuclear protein) and PA (polymerase) differ in their phenotypic and functional signature, including CD103 expression [39]. In addition, NP- and PA-specific effector cells show a different ability to differentiate into memory cells during resolution of acute infection [40]. This is due to a differential nature and kinetic of DC antigen-presentation of NP and PA antigens [40]. In a mouse model of hepatitis B virus (HBV) infection, CD8+ T cells specific for different epitopes can show distinct antiviral efficacies [41]. Recent studies have also shown that in acute and chronic human HBV infection, CD8+ T cells specific for HBV core or polymerase epitopes differ in phenotype and functionality [42,43]. The causes and the functional effects of this heterogeneity in the HBV epitope-specific CD8+ T cell response are currently unclear. However, recent work on human liver parenchyma and in vitro systems showed spatiotemporal differences in the presentation of distinct MHC class I HBV epitopes by hepatocytes [44]. This may contribute to the formation of the observed epitope-specific differences in the CD8+ T cell response. Future studies, using the NrHV mouse model and NS3+ and NS4+ CD8+ T cells may help to determine if factors such as differential modes of DC antigen-presentation, TCR signal strength, or spatiotemporal differences in antigen-presentation by infected cells can contribute to CD8+ T cell epitope-heterogeneity during a hepatic viral infection in vivo.

Interestingly, our functional analysis of major CD8 effector molecules reveled that neither cytokine-mediate (IFN-γ, TNF-α) or cytotoxic functions (Perforin) play a dominant role in NrHV clearance. These findings suggest that the establishment of chronic hepacivirus infection in the absence of CD4+ T cell help is likely associated with a multi-layered impairment of effector CD8+ T cell responses during acute infection.

Indeed, our parallel analysis of CD4-helped and un-helped NS3- and NS4-specific CD8+ T cells showed multiple phenotypical and functional differences. We observed a loss of short-lived effector cells, downregulation of key transcription factors for cytotoxic function, upregulation of inhibitory receptors and decreased polyfunctionality in CD8+ T cells lacking CD4 help. A similar functional impairment of CD8+ T cells was observed in mice receiving CD40L blocking antibody early during NrHV infection. This finding shows that CD40L-CD40 interactions are an important mechanism of CD4+ T cell help for CD8+ T cells during NrHV infection. Overall, our data are in line with numerous other studies characterizing helped versus un-helped CD8+ T cells in various infection models [13,45–48].

Of note, we had previously shown that long-term chronic NrHV infection is associated with T cell exhaustion, such as high PD-1 expression [18]. Another study recently extended the analysis of CD8+ T cells during long-term chronic infection. This study showed that multiple inhibitory receptors are highly expressed early on during chronic infection and associated with limited cytokine production and functional impairment. The results confirm T cell dysfunction in chronic NrHV infection similar to chronic HCV infection in humans [5,22].

An unexpected finding of our study was the induction of a CD103+CD49a+ effector subset, which was enriched in the NS3-specific population and specifically lost or not induced in the absence of CD4+ T cell help and CD40L-CD40 interactions. CD103 and CD49a expression are mostly associated with Trm cells [8]. While human Trm cells in the liver express CD103, it has been shown that pathogen-specific Trm cells in the mouse liver usually do not express CD103 [24]. However, studies on inflammatory liver diseases, such as primary biliary cholangitis, report CD103+ Trm cells in the mouse liver [49]. Therefore, CD103 expression on liver CD8+ T cells might be context dependent.

We detected increased serum TGF-β levels during acute infection. TGF-β induces the expression of both CD103 and CD49a, which may explain the emergence of CD103+CD49a+ cells during NrHV infection [8,9]. Interestingly, we observed that a lack of CD4+ T cells reduced serum TGF-β levels. While this was not reflected in liver-tissue TGF-β levels at the time point analyzed, we detected significantly reduced tissue IL-15 levels. IL-15 is another essential cytokine for the induction and maintenance of tissue-resident CD8+ T cells [33]. This could indicate that the loss of CD103+CD49a+ CD8+ T cells is linked to decreased tissue IL-15 levels in the absence of CD4+ T cell help. Additional studies are needed to define the source of TGF-β and IL-15 and its impact on the T cell population during NrHV infection.

We detected CD103 and CD49a expression in CD127- effector and CD127+ memory precursor subsets at day 14 and 21 of acute infection, but a lack of CD4+ T cell help mostly reduced CD103+CD49a+ effector cells at these time points. For that reason, we focused our analysis on the effector subset. However, CD8+ T cell differentiation during viral infection is highly heterogenous and plastic and we cannot exclude that these effector cell may develop into memory cells during viral resolution [7]. Further, it is unfortunately not possible to assess memory formation in the absence of early CD4+ T cell help since mice depleted of CD4+ T cells at the early stages develop chronic infection.

CD103+CD49a+ effector cells were enriched in the liver as compared to the peripheral blood. Using intravascular staining, we found that CD103+CD49a+ cells were present at equal percentages in the intravascular and extravascular space, while CD103-CD49a+ cells were highly enriched in hepatic vasculature. Recent studies have shown that liver-resident cells, unlike resident cells in other organs, often remain in the vascular space rather than migrating into the tissue [29,30]. It will be interesting to further investigate the location of different effector and memory T cell subsets in the NrHV mouse models using techniques such as intravital microscopy.

Most relevant for our present study, a lack of CD4+ T cell help resulted in a general loss of the CD103+CD49a+ subset, and to a lesser extent the CD103-CD49a+ subset, from all liver compartments and the peripheral blood. Interestingly, the CD103+CD49a+ subset showed an elevated capacity to produce TNF-α and IFN-γ. This is in line with a recent study showing increased cytokine production by CD103+CD49a+ influenza-specific Trm cells [50]. Adoptive transfer of sorted CD103+/-CD49a+ cells showed that these cells, at low numbers, can directly mediate NrHV clearance in some mice. Additional experiments using higher numbers of transferred cells will help to further investigate the direct role of these cells in vivo. Together, our data indicate that the loss of a liver-homing highly functional effector CD103+CD49a+ subset might be a relevant factor contributing to the establishment of chronic infection in the absence of CD4+ help.

Our study was primarily focused on the impact of CD4+ T cell help on effector CD8+ T cells during acute infection. However, since CD103 and CD49a expression are linked to Trm cells, we analyzed if CD103+CD49a+ cells persisted in the liver as memory cells after viral resolution. We found that the CD103+CD49a+ population is maintained as a long-term CD69+CD38+CXCR6+ Trm subset in the liver, which showed rapid expansion and cytokine production after secondary infection. CD69 is an important marker of tissue-residency. Further, it has recently been shown that CD38 and CXCR6 expression defines Trm cells in multiple organs and during different types of infection [28]. As mentioned above, our study could not determine if the CD103+CD49a+ Trm subset is derived from the effector population that is lost with the lack of CD4+ help or if other memory precursor subsets gives rise to the CD103+CD49a+ Trm subset. Future studies using lineage tracing and adoptive transfer experiments may help to address this question.

Our study shows that a lack of CD4+ T cells or CD40L signaling have a similar impact on the CD8+ T cell response, both leading to reduced expression of effector functions and a loss of the CD103+CD49a+ subsets. These findings suggest a role of CD4+ T cell mediated activation of DCs through CD40L-CD40 interactions and DC cross-presentation to CD8+ T cells in NrHV infection. However, follow-up studies need to assess mechanisms of CD4+ T cell help, DC antigen-presentation and T cell priming in more detail. This is of particular importance for a hepatic virus infection since the liver provides a unique microenvironment that allows T cell priming directly in the liver as opposed to the lymph node (LN) [31]. Interestingly, a recent study using adeno-associated viral vector mediated antigen-expression specifically in hepatocytes showed that CD4+ T cells use CD40L-CD40 interactions with hepatic DCs to promote CD8+ T cell responses [51]. The NrHV mouse model represents a valuable model to investigate CD4-DC-CD8 interactions and LN versus hepatic CD8+ T cell priming during a true hepatic virus infection in vivo.

Taken together, our study demonstrates that early CD4+ T cell help, by mechanisms that include CD40L signaling, is critical for acute hepacivirus clearance. We show that the generation of a functional hepacivirus-specific effector CD8+ T cell response is help-dependent. A lack of early CD4+ T cell help leads to a multifaceted impairment of the effector response, including the loss of a liver-homing CD103+CD49a+ subset. Further, NrHV-specific CD103+CD49a+ cells represent a long-term hepatic Trm population. Our study provides new insights into the hepatic T cell response during hepacivirus infection and may have implications for HCV vaccine strategies.

## Material and methods

### Ethics statement

All mice were bred and maintained under specific pathogen-free conditions at the Institute for Animal Studies of Albert Einstein College of Medicine. All experiments with mice were conducted in accordance with the NIH Guide for the Care and Use of Laboratory Animals and approved by the Albert Einstein College of Medicine Institutional Animal Care and Use Committee (protocol number 00001108).

### Mice

C57BL/6 and B6.Cd8^-/-^, B6.Tnf-α^-/-^, B6.Prf^-/-^, B6.Ifnyr^-/-^ mice were purchased from the Jackson Laboratory. Knockout mice used in experiments were bred in-house and wild-type mice were purchased from Jackson Labs. Mice were fed ad libitum with a regular chow. For acute infection experiments 8–10 week-old female and male mice were used. For memory and reinfection experiments, age-matched 7–12 month-old mice were used after having been initially infected at 8 weeks old. All experiments in this study were performed using 4–8 mice per group. Mice were on a 12-hour day-night cycle and experiments and dissections were performed during the day cycle.

### Virus infection

The Norway rat hepacivirus (NrHV) inoculum used in this study was a cDNA clone-derived virus stock as described [19]. Mice were infected intravenously with 10^4^ NrHV genome equivalents (GE) by retro-orbital injection.

### Viral RNA isolation and RT-qPCR

Viral RNA from mouse serum was isolated using the High Pure Viral Nucleic Acid Kit (Roche). NrHV RNA was detected and quantified by one-step RT-qPCR using TaqMan Fast Virus 1-Step Master mix (Applied Biosystems) on Quantstudio 6 flex (Applied Biosystems) with the following protocol: 50°C for 30 min, 95°C for 5 min followed by 40 cycles of 95°C for 15 sec, 56°C for 30 sec and 60°C for 45 sec.

An NrHV standard curve was generated from *in vitro* transcribed RNA from a plasmid encoding the partial NrHV E1 or NS3 sequence. After *in vitro* transcription, the RNA was quantified and diluted to concentrations ranging from 10^8^−10^1^ GE/μl.

The sequences of NrHV E1 specific primers used for this protocol were:

Sense: GGCTGTGTCATCTGCGAGCA,

Anti-sense: CGACGAAGTCTATATGGTGGGC

Probe: [6-FAM]GGCCCCATGGTATCCAGGTCACCGCACTA[BHQ1a-6FAM]

The sequences of NrHV NS3 specific primers used for this protocol were:

Sense: TACATGGCTAAGCAATACGG

Anti-sense: AAGCGCAGCACCAATTCC

Probe: [6-FAM]CTCACGTACATGACGTACGGCATG[BHQ1a-6FAM]

### Isolation of lymphocytes

Mice were sacrificed and dissected at multiple time points post-infection (pi) and leukocytes were immediately isolated from blood and liver. Blood-derived leukocytes were isolated from heparinized blood by Ficoll-density gradient (Corning Lymphocyte separation medium) centrifugation (20min, 2000rpm, 20°C). The liver tissue was minced and digested with a digestion buffer (composed of HBSS, 0.01% collagenase IV, 40mM HEPES, 2mM CaCl_2_ and 2U/ml DNAse I) for 20min at 37°C followed by homogenization through a 100μm cell strainer. Leukocytes were subsequently isolated from liver cell suspension by Ficoll-density gradient centrifugation.

### Flow cytometry

Lymphocytes isolated from liver and blood at several time points pi were plated in 96 well V bottom plates in DPBS. An initial viability stain (Zombie Nir Fixable Viability Kit, BioLegend) was performed. Tetramer staining was performed in staining buffer (DPBS with 1% FBS) at 37°C for 15 minutes prior to surface staining. For this study, we used NrHV NS3_1043-1052_ (CTEFYLATRL) and NS4_1602-1611_ (SAALNPAPEM) MHC class I tetramers as described [20]. Surface antibody staining was performed at 20°C for 15 minutes in staining buffer. For experiments involving intracellular staining, cells were permeabilized (eBioscience FOXP3/Transcription Factor Staining Buffer Set) for 20 minutes at 4°C and then stained for intracellular factors for 30 minutes at 4°C. After staining was completed, all cells were fixed with 4% paraformaldehyde (PFA). FACS analysis was performed using Cytek Aurora analyzer (Cytek). Spectroflow (Cytek) and Flow Jo Version 10 (BD Biosciences) software were used for data analysis. FMO (fluorescence minus one) controls for major antibodies and MHC class I tetramers used in this study are show in S2 Fig.

### Intravascular staining

Intravascular staining of CD8+ T cells was performed as described [32]. Specifically, intravascular staining was performed by intravenous injection of 4μg anti-CD8 antibody into mice four minutes prior to sacrifice and dissection. Following dissection, ex vivo staining with another anti-CD8 antibody (of the same clone, but distinct fluorophore) was performed.

### Cytokine analysis

Isolated lymphocytes were plate in 96 well plates in RPMI culture medium for 12 hours at 37°C. Cytokine analysis of various CD49a/CD103 subsets and TGFβ/LAP expression by CD4+ T cells was performed immediately after isolation without incubation.

Lymphocytes were stimulated with the NrHV NS3 and NS4 peptides corresponding to the MHC class I tetramers, culture media alone as a negative control, or 50ng/mL of PMA and 500ng/mL Ionomycin as a positive control. Stimulation was performed in the presence of Brefeldin A. Antibody staining for CD107a was performed during the 5-hour stimulation period and all other staining was performed afterwards.

### Cytokine/Tetramer co-analysis

Hepatic lymphocytes isolated from mice at day 21 pi and uninfected control mice were stimulated as described above with NrHV peptides. Antibody staining of all surface and intracellular markers was performed after permeabilization. MHC class I tetramer staining was performed for 60 minutes and during the last twenty minutes surface and intracellular antibodies were added to the staining mixture. After staining samples were fixed with 4% PFA.

### CD4+ T cell depletions

CD4 depletion was performed using 400μg of anti-CD4 antibody (GK1.5/bioxcell) injected intraperitoneally. For experiments determining the timing of the role of CD4 T cells, groups of mice were injected with 400μg of anti-CD4 antibody at a single time point (days:-4,0,3,6,9,7,8,12,15,18 pi) and NrHV viremia was examined throughout the course of infection in comparison to a non-depleted control group. For flow cytometry experiments examining the impact of CD4 depletion on CD8+ T cells at day 14 and day 21 pi, groups of mice were depleted of CD4 T cells 4 days prior to infection.

### Adoptive T cell transfer

Lymphocytes were isolated as previously stated from NrHV infected C57BL/6 wild type mice dissected at day 14 pi. Following isolation cells were stained with surface antibodies allowing for the identification of CD8+ T cells. Prior to flow assisted cell sorting, cells were stained with DAPI to determine cell viability. All cells were sorted using a BD Aria cell sorter. CD8+ T cells were then immediately transferred into B6.Cd8^-/-^ mice at day 14 pi. In experiments that involved the transfer of all CD8+ T cells at day 14 pi, each mouse received 6.9x10^4^ sorted CD8+ T cells. 3.45 x10^4^ cells were injected intravenously (i.v.) and 3.45x10^4^ cells were injected intraperitoneally (i.p.). In experiments that involved the transfer of all CD8+ T cells at day 7 pi, each mouse received 1.65x10^4^ sorted CD8+ T cells. 8.25 x10^3^ cells were injected intravenously (i.v.) and 8.25x10^3^ cells were injected intraperitoneally (i.p.). The control group in each experiment was injected with DPBS. Serum was collected weekly to measure NrHV viremia over time. In experiments that involved the transfer of CD49a and CD103 subsets of CD8+ T cells each mouse received 6x10^3^ effector (CD127-) CD8+ T cells. 3x10^3^ cells were injected intravenously (i.v.) and 3x10^3^ cells were injected intraperitoneally(i.p.).

### Neutralization experiments

CD40L neutralization experiments were performed by i.p. injection of 200μg anti-CD40L antibody (MR-1 / bioxcell) on day 3 and 5 pi. Control mice were injected with 200μg of an isotype control antibody on the same days (polyclonal Armenian hamster IgG / bioxcell).

IFN-γ and TNF-α dual neutralization experiments were performed by i.p. injection of 500μg anti IFN-γ antibody (XMG1.2 / bioxcell) and 500μg anti TNF-α antibody (XT3.11 / bioxcell) on days 12,15,18,21 pi.

### Total serum IgG analysis

Serum IgG levels from mice that received either anti-CD40L antibody or isotype control (as described above; hamster IgG) were determined using Invitrogen’s Mouse IgG (Total) ELISA kit following the provided protocol.

### Serum TGF-beta analysis

Serum TGF-beta levels were determined using Invitrogen’s Mouse TGF-beta 1 ELISA kit. Work was performed according to manufacturer’s instructions.

### Liver tissue protein isolation

Liver tissue samples were taken from the largest lobes of uninfected control mice, infected mice at day 14 pi and CD4+ depleted mice at day 14 pi. Liver samples were flash frozen in a dry ice and ethanol bath for 5 minutes and then transferred into -80°C until protein extraction was performed. Liver samples were weighed and then placed in protein lysate buffer (Tissue Extraction Reagent I (Invitrogen), 1 mM PMSF (Invitrogen), Pierce Protease Inhibitor Mini Tablets (Invitrogen) and mechanically homogenized. Homogenized samples were centrifuged at 13000g at 4°C for 20 minutes and supernatant was collected, aliquoted and frozen at -80°C until analysis.

### Liver IL-15 analysis

IL-15 levels from homogenized liver lysates (see above) were obtained using PeproTech Murine Il-15 standard ABTS ELISA Development Kit following the provided protocol.

### Liver TGF-beta analysis

TGF-beta levels from homogenized liver lysates were obtained using Invitrogen Mouse TGF beta1 ELISA Kit following the provided protocol.

### t-SNE analysis

FlowJo’s concatenation and t-SNE features were used to construct the t-SNE graphs displayed throughout the paper. The t-SNE shown in Fig 2 was created by concatenating hepatic CD8+ T cells using FlowJo’s concatenate sample from 6 samples from each timepoints of interest, ensuring that each timepoint had an equal number of CD8+ T cells from each timepoint and sample. The number of events in the t-SNE analysis were 92592. The markers used to construct the t-SNE were EOMES, CD62L, KLRG-1, CD103, LY108, T-bet, ki67, CX3CR1, CD69, CD127, TOX, PD1, CD44, TIM3, TCF1. The TSNE was constructed using opt-SNE approach with the default settings as follows: 1000 iterations, a perplexity rating of 30, a learning rate of 6481, using an exact vantage point tree KNN algorithm and a Barnes-Hut gradient algorithm. After constructing the t-SNE, gating was performed first on our two Tetramers NS4 and NS3. Then gating was performed on CD127 and KLRG1 expression and overlayed on the t-SNE to show the distribution of different memory and effector populations.

The t-SNE used in Figs 4 and 5 was used the same methodology as the t-SNE in figure two except for the fact that each group had 6 mice from the same timepoint of infection (D21), but having been under different conditions (either a control group or a group that was CD4 depleted). The learning rate was 9221. The method of concatenation and gating were identical to the previous strategy. 131736 events were included in this t-SNE.

Single marker expression heatmaps of the t-SNE shown in Figs 2 and 4 are shown in S10–S11 Figs.

### Statistical analysis

Statistical analysis was performed using GraphPad Prism (Version 10). ANOVA, paired and unpaired T tests were performed as appropriate. Statistical analysis was not based on sex of mice.

All antibodies and reagents are listed in S1 Table.

## Supporting information

S1 Fig (A) WT C57BL/6 mice were depleted of CD4+ T cells during ongoing NrHV infection (day 7 and day 9 pi). CD4+ T cell levels in the peripheral blood were analyzed directly prior to depletion and 5 to 7 days after depletion. (B) NrHV viremia in mice depleted of CD4+ T cells starting day 7 and day 8 pi. (C) CD8^-/-^ mice from experiment shown in Fig 1D were analyzed for the presence of hepatic CD8+ T cells 3 months after adoptive CD8+ T cell transfer. Mice were reinfected with NrHV and 7 days post reinfection CD8+ T cell levels were analyzed in mice that never received CD8+ T cells (PBS control) and mice that initially received CD8+ T cells (CD8+ T cell transfer). (D) A similar CD8+ T cell transfer experiment as described in Fig 1C–1D in additional groups of mice. CD8+ T cells were transferred on day 7 pi. (E) Total serum IgG levels were analyzed by ELISA in control mice (control isotype antibody: day 3 and 5 pi) and CD40L blocked mice (anti-CD40L: day 3 and 5 pi) at day 0 and day 35 pi. Graphs show individual mice or mean with SD. LOQ: limit of quantification. Statistics: unpaired two-tailed t test; * p<0.05.
(TIF)

S2 Fig (A) Gating strategy for hepatic CD8+ T cell effector and memory subsets based on CD44, CD127 and KLRG-1 expression. (B) Representative FACS plots for CD103, T-bet, ki67, Eomes, CX3CR1 and TCF-1 expression in hepatic CD8+ T cells. (C) FMO (fluorescence minus one) controls for indicated antibodies and NS3- and NS4-specific tetramers.
(TIF)

S3 Fig Expression levels of TCF-1 (A), CX3CR1 (B) and ki67 (C) on NS3- and NS4-specific CD8+ T cell subsets at indicated time points (n = 6). Graphs show mean with SD. Statistics: two-way ANOVA with Tukey multiple comparison test; * p<0.05, ** p<0.01, *** p<0.001, **** p<0.0001.
(TIF)

S4 Fig (A) Original FACS plots showing the co-expression of IFN-γ and TNF-α with CD107a of hepatic CD8+ T cell with no stimulation (negative control), after unspecific stimulation with PMA/Ionomycin or stimulation with the NS3 or NS4 peptides at day 21 pi. (B-D) Tetramer/cytokine co-staining of hepatic NS4+ CD8+ T cells. (B) Frequency of detectable NS4+ cells with the co-staining method at day 21 pi as compared to uninfected negative control (left graph). Percentage of CD107a+, IFN-γ+ and TNF-α+ cells within the NS4+ population (right graph) (n = 4). (C) Original FACS plots showing NS4-tetramer staining with the co-staining method at day 21 pi and in an uninfected control mouse. The NS4 FMO (Fluorescence Minus One) control is also shown. (D) Original FACS plots showing CD107a+, IFN-γ+ and TNF-α+ cells within the NS4+ population at day 21 pi and in all CD8+ T cells in the uninfected control.
(TIF)

S5 Fig (A) Expression of Eomes and T-bet and in general (all CD8), NS3- and NS4-specific CD8+ T cell subsets in CD4-depleted mice and controls at day 21 pi (n = 6). (B) LAG-3, Ly108, PD-1 and Tim3 expression by NS3- and NS4-specific effector cells in the presence or absence of CD4 + T cell help. (C) Total hepatic cell numbers of all CD8+ T cells, NS3- and NS4-specific cells. (D) Expression of Eomes and T-bet and in general (all CD8), NS3- and NS4-specific CD8+ T cell subsets in CD40L blocked mice and controls at day 21 pi (n = 6). (E) LAG-3, Ly108, PD-1 and Tim3 expression by NS3- and NS4-specific effector cells in the presence or absence of CD40L interactions. (F) Expression of CD103 in general (all CD8), NS3- and NS4-specific CD8+ T cell subsets in CD4-depleted mice and controls at day 21 pi. Graphs show mean with SD. Statistics: unpaired two-tailed t test; * p<0.05, ** p<0.01, *** p<0.001, **** p<0.0001.
(TIF)

S6 Fig (A) Representative FACS plots showing polyfunctional (IFN-γ+TNF-α+CD107a+) effector functions after stimulation with the NS4 peptide or PMA/Ionomycin in CD8+ T cells from control mice, CD4-depleted mice and CD40L blocked mice. (B) Representative FACS plots (from concatenated samples from each group) showing LAG-3, PD-1, Ly108, TIM-3 expression on NS3- and NS4-specific CD8+ T cells from CD4-depleted mice and controls.
(TIF)

S7 Fig (A) Total hepatic cell numbers of all CD8+ T cells, NS3- and NS4-specific cells. (B) Frequencies of CD103/CD49a subsets within the NS3- and NS4-specific CD8+ T cell populations at day 14 and 21 pi in CD4-depleted mice and controls (n = 4–6). (C) CD49a and CD103 expression on liver and peripheral blood derived NS3- and NS4-specific effector cells at day 14 and 21 pi in WT control mice (n = 6). (D) Percentages of CD103/CD49a subsets within the NS3-specific populations from liver IVAb+, liver IVAb- and peripheral blood at 21 pi in CD4-depleted mice and controls (n = 6). (E) Adoptive transfer of sorted CD49a+CD103+, CD49a+CD103-, CD49a-CD103 populations (6x10^3^ cells/mouse, n = 4) or total CD8+ T cells (6.9x10^4^ cells/mouse, n = 7), or PBS (n = 6) into congenic CD8 knock-out (KO) mice at day 14 pi. Experimental outline similar to Fig 1D. Graphs show mean with SD or data points of individual mice. LOQ: limit of quantification. Statistics: unpaired two-tailed t test or one-way ANOVA with Tukey multiple comparison test; * p<0.05, ** p<0.01, *** p<0.001, **** p<0.0001.
(TIF)

S8 Fig (A) Frequencies of NS3- and NS4-specific cells in the liver IVAb+, liver IVAb- compartment and peripheral blood at day 14 and 21 pi (n = 6). (B) Serum TGF-β levels at day 7 and day 14 pi (n = 8 per group). (C) Tissue TGF-β and IL-15 levels at day 14 pi (n = 4). (D) Flow cytometric analysis of TGF-β/LAP expression by hepatic CD4+ T cells at day 14 pi and in uninfected controls (n = 4). Left: total hepatic cell numbers. Right: Original FACS plot from day 14 pi as compared to FMO control. (E) CD38, CD69, CXCR6, LFA-1, HIF-2a and TCF-1 expression in the respective NS4+ memory CD103/CD49a subsets. (F) MFI (mean fluorescence intensity) of HIF-2a and LFA-1 in NS3+ and NS4+ memory CD103/CD49a subsets. Statistics: unpaired two-tailed t test or one-way ANOVA with Tukey multiple comparison test; * p< 0.05, ** p< 0.01, *** p<0.001, **** p<0.0001.
(TIF)

S9 Fig A) Percentages of NS4+ CD44+ CD8+ T cells in the liver and blood of memory mice or at 5 days post reinfection (n = 4–8). (B) Percentages of hepatic NS4+ CD49a/CD103 subsets from memory and reinfection mice(n = 4–8). (C) Total hepatic cell count of NS4+ CD49a/CD103 subsets. Graphs show mean with SD (n = 4–8). (D) FACS plots from representative samples of NS4+ CD49a/CD103 cells of memory and reinfection mice. (E) TNF-α, IFN-γ and/or CD107a expression of hepatic CD103/CD49a CD8+ T cell subsets after unspecific stimulation with PMA/Ionomycin at day 5 of secondary infection.
(TIF)

S10 Fig Single expression heatmaps for markers used in the t-SNE shown in Fig 2D. Heatmaps are shown for the “all” t-SNE. (TIF)

S11 Fig Single expression heatmaps for the markers used in the t-SNE shown in Fig 4C. Heatmaps are shown for the “combined” t-SNE. (TIF)

S1 Table Materials list divided into several tables. (A) Fluorescently labeled antibodies used for flow cytometry. (B) Antibodies used for in vivo work. (C) Non-antibody reagents used (D) Commercial Assay Kits. (E) Specialized equipment. (F) Viral peptide identities and sequence. (G) Mouse strains and origin. (H) Fluorescently labeled tetramers and the sequence of their bound peptides. (I) Software used in data analysis and figure design.
(XLSX)
