# Supplementary figures and images for: CD4+ T cell help during early acute hepacivirus infection is critical for viral clearance and the generation of a liver-homing CD103+CD49a+ effector CD8+ T cell subset
Source: PLoS Pathog. 2024 Oct 11;20(10):e1012615. doi: 10.1371/journal.ppat.1012615 (PMC11498735; doi:10.1371/journal.ppat.1012615)

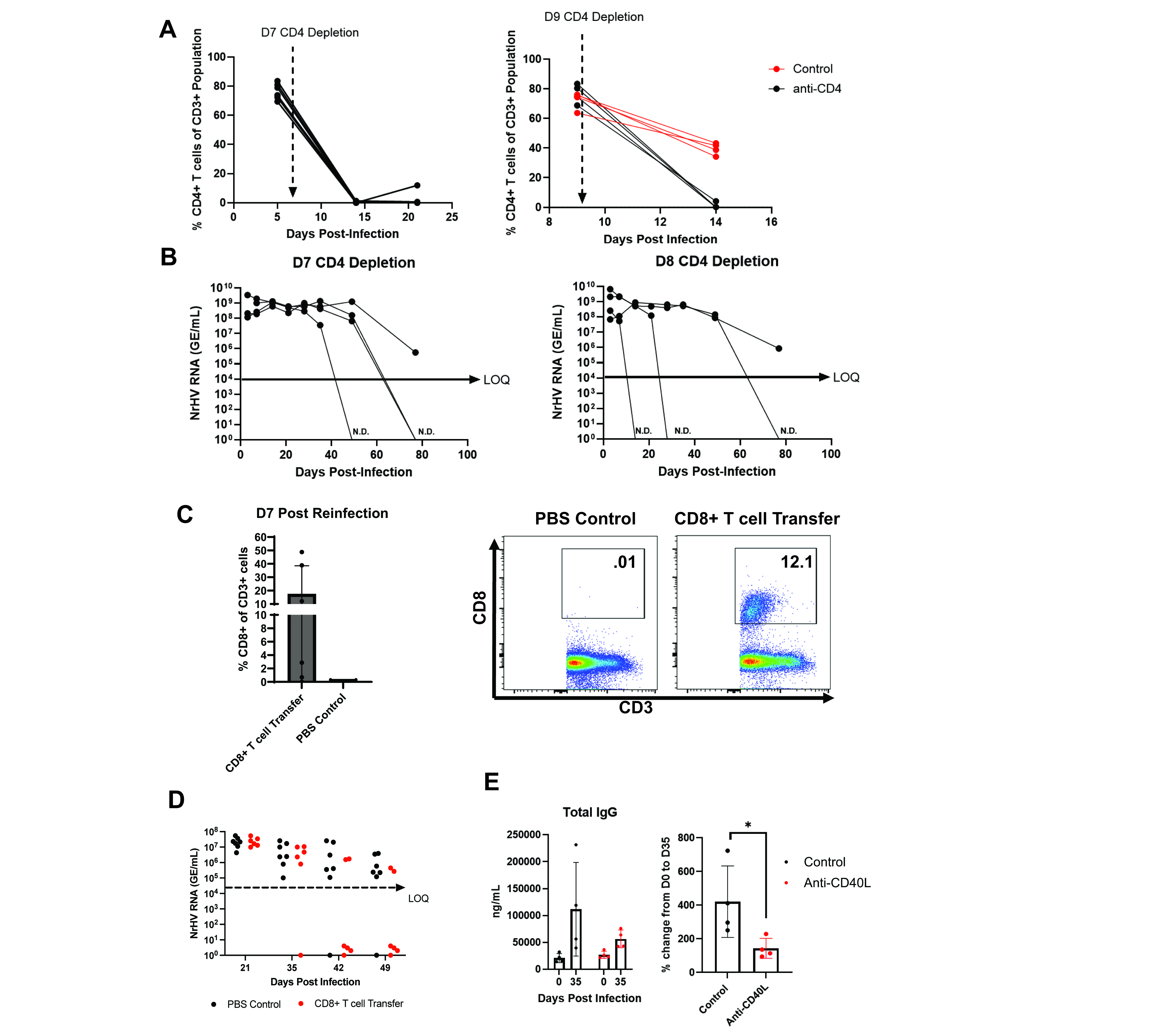

Supplement: S1 Fig — (A) WT C57BL/6 mice were depleted of CD4+ T cells during ongoing NrHV infection (day 7 and day 9 pi). CD4+ T cell levels in the peripheral blood were analyzed directly prior to depletion and 5 to 7 days after depletion. (B) NrHV viremia in mice depleted of CD4+ T cells starting day 7 and day 8 pi. (C) CD8-/- mice from experiment shown in Fig 1D were analyzed for the presence of hepatic CD8+ T cells 3 months after adoptive CD8+ T cell transfer. Mice were reinfected with NrHV and 7 days post reinfection CD8+ T cell levels were analyzed in mice that never received CD8+ T cells (PBS control) and mice that initially received CD8+ T cells (CD8+ T cell transfer). (D) A similar CD8+ T cell transfer experiment as described in Fig 1C–1D in additional groups of mice. CD8+ T cells were transferred on day 7 pi. (E) Total serum IgG levels were analyzed by ELISA in control mice (control isotype antibody: day 3 and 5 pi) and CD40L blocked mice (anti-CD40L: day 3 and 5 pi) at day 0 and day 35 pi. Graphs show individual mice or mean with SD. LOQ: limit of quantification. Statistics: unpaired two-tailed t test; * p<0.05. (TIF) [file ppat.1012615.s001.tif]

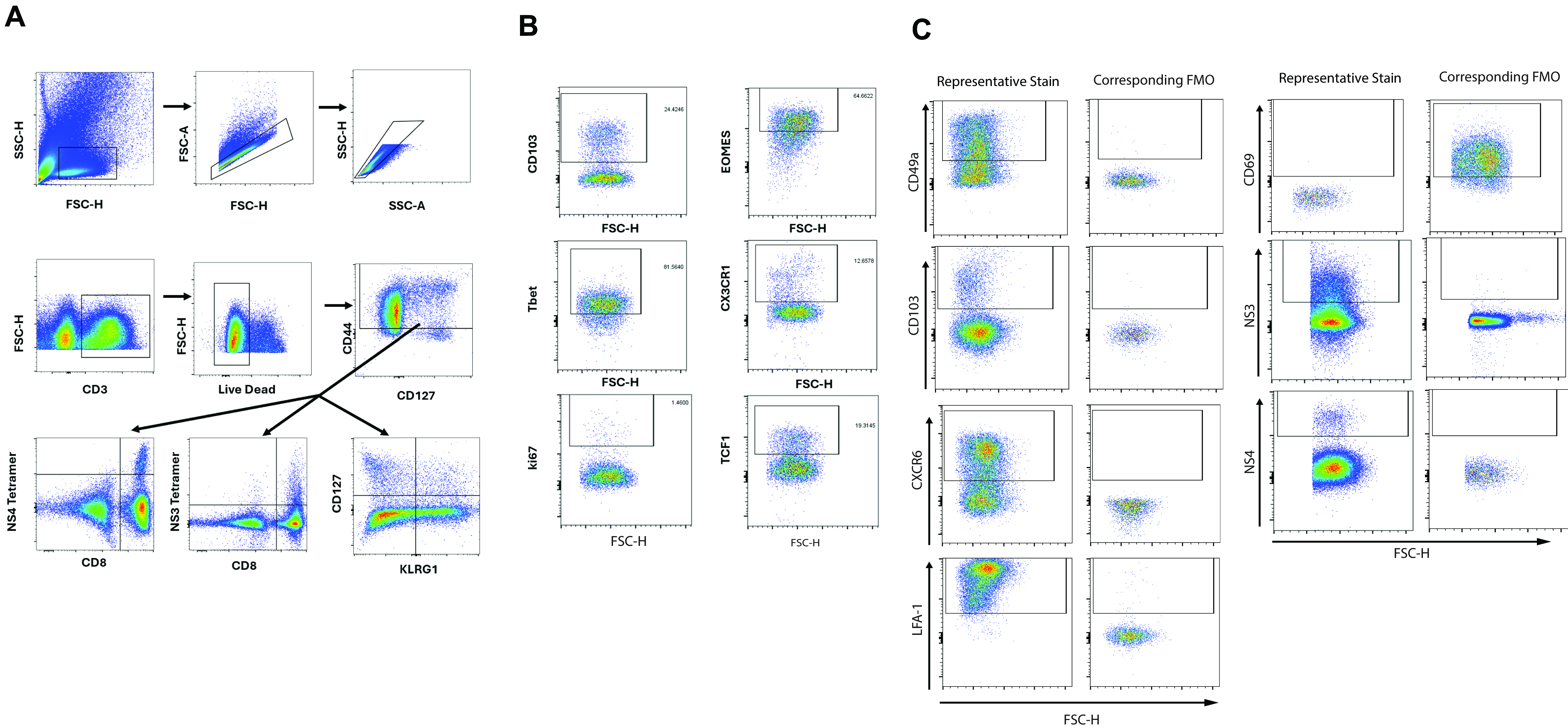

Supplement: S2 Fig — (A) Gating strategy for hepatic CD8+ T cell effector and memory subsets based on CD44, CD127 and KLRG-1 expression. (B) Representative FACS plots for CD103, T-bet, ki67, Eomes, CX3CR1 and TCF-1 expression in hepatic CD8+ T cells. (C) FMO (fluorescence minus one) controls for indicated antibodies and NS3- and NS4-specific tetramers. (TIF) [file ppat.1012615.s002.tif]

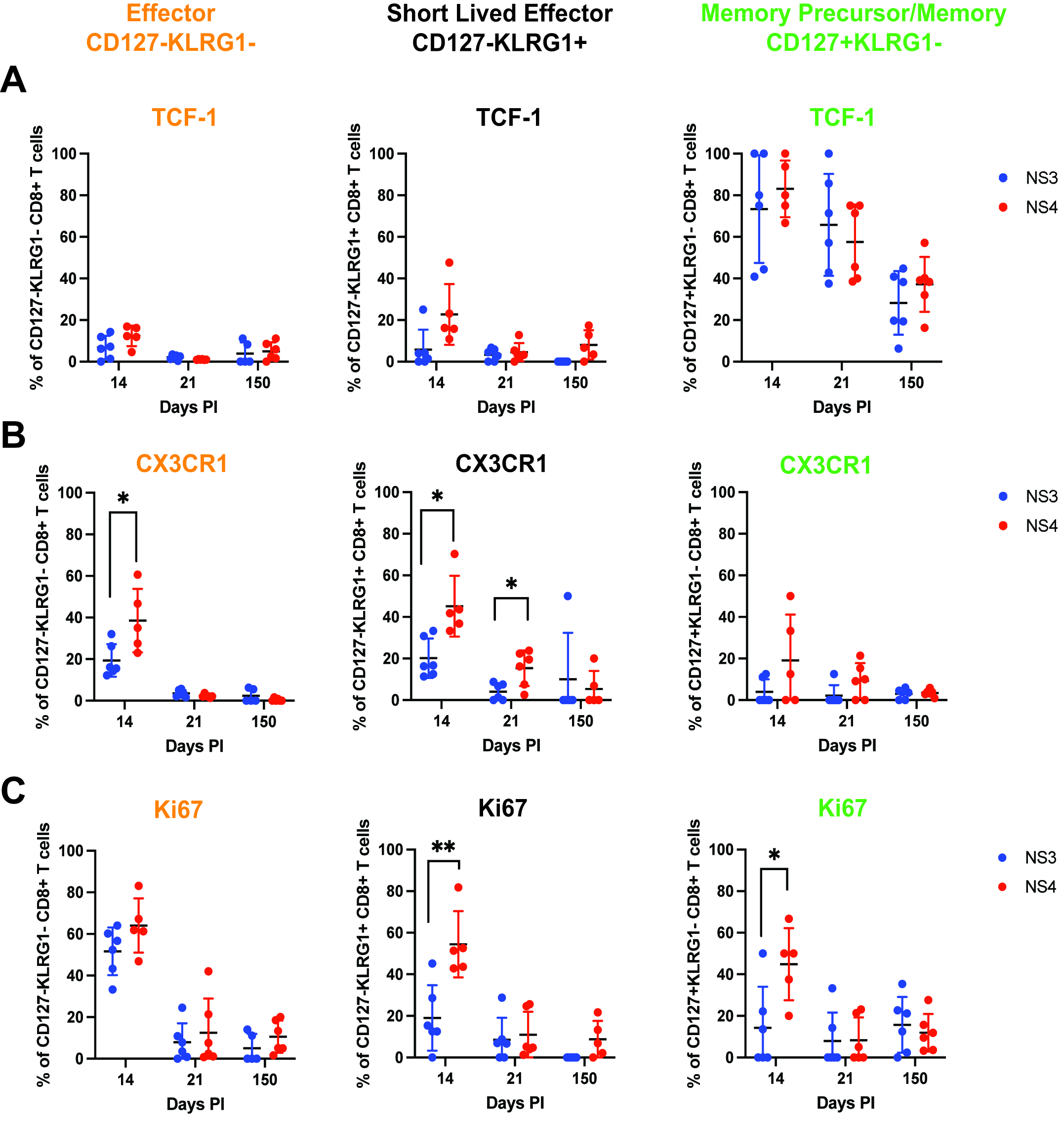

Supplement: S3 Fig — Expression levels of TCF-1 (A), CX3CR1 (B) and ki67 (C) on NS3- and NS4-specific CD8+ T cell subsets at indicated time points (n = 6). Graphs show mean with SD. Statistics: two-way ANOVA with Tukey multiple comparison test; * p<0.05, ** p<0.01, *** p<0.001, **** p<0.0001. (TIF) [file ppat.1012615.s003.tif]

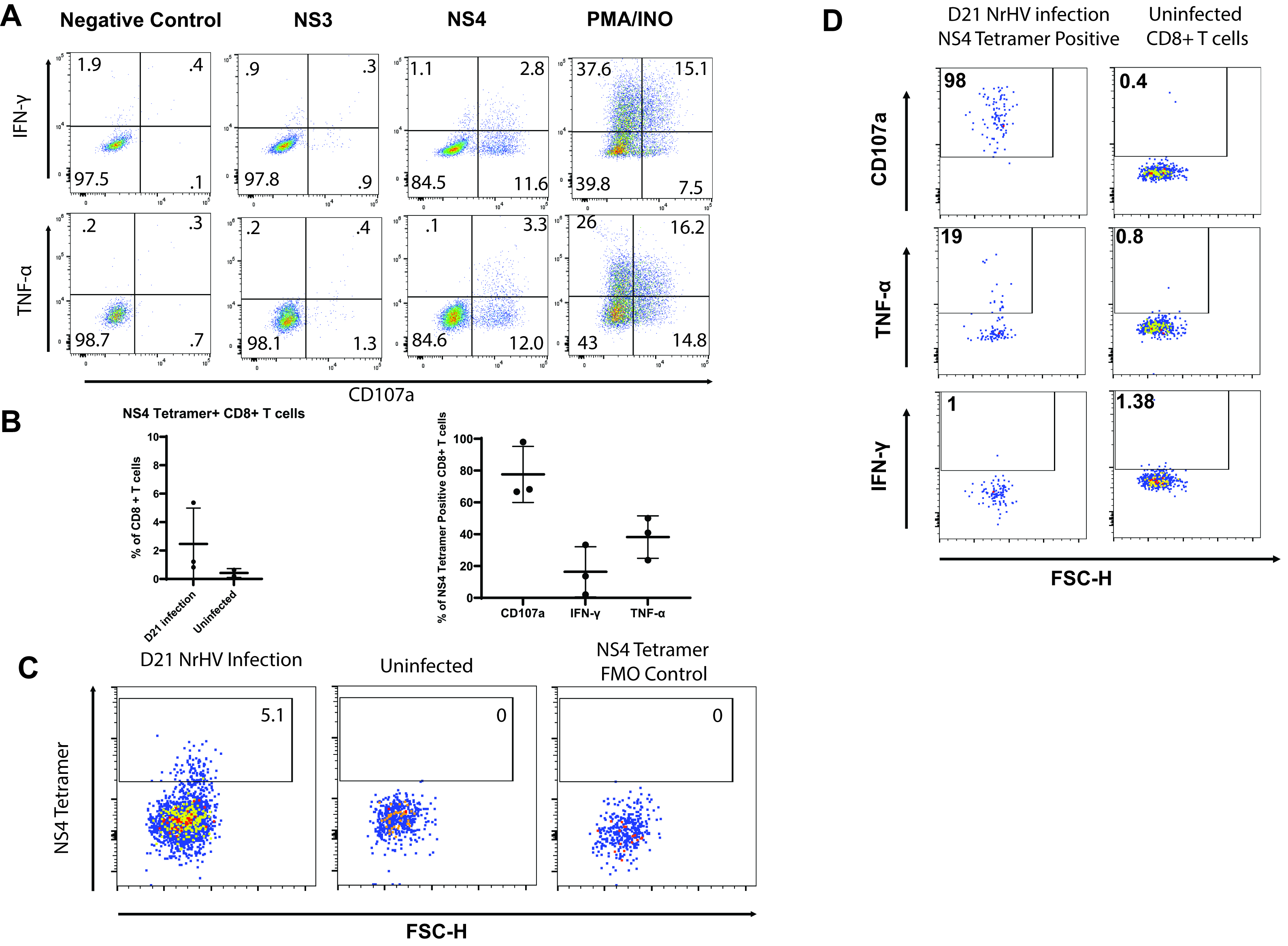

Supplement: S4 Fig — (A) Original FACS plots showing the co-expression of IFN-γ and TNF-α with CD107a of hepatic CD8+ T cell with no stimulation (negative control), after unspecific stimulation with PMA/Ionomycin or stimulation with the NS3 or NS4 peptides at day 21 pi. (B-D) Tetramer/cytokine co-staining of hepatic NS4+ CD8+ T cells. (B) Frequency of detectable NS4+ cells with the co-staining method at day 21 pi as compared to uninfected negative control (left graph). Percentage of CD107a+, IFN-γ+ and TNF-α+ cells within the NS4+ population (right graph) (n = 4). (C) Original FACS plots showing NS4-tetramer staining with the co-staining method at day 21 pi and in an uninfected control mouse. The NS4 FMO (Fluorescence Minus One) control is also shown. (D) Original FACS plots showing CD107a+, IFN-γ+ and TNF-α+ cells within the NS4+ population at day 21 pi and in all CD8+ T cells in the uninfected control. (TIF) [file ppat.1012615.s004.tif]

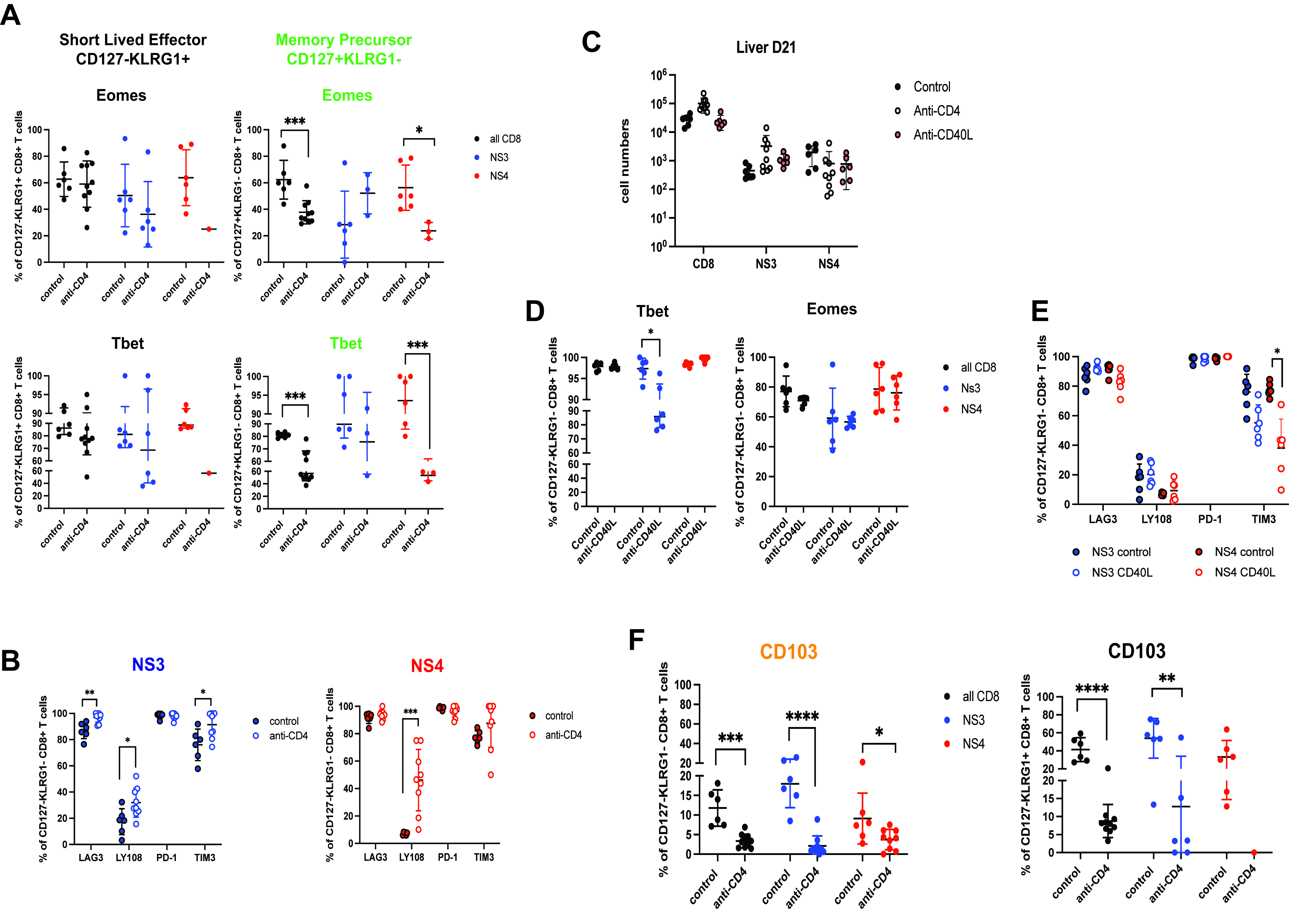

Supplement: S5 Fig — (A) Expression of Eomes and T-bet and in general (all CD8), NS3- and NS4-specific CD8+ T cell subsets in CD4-depleted mice and controls at day 21 pi (n = 6). (B) LAG-3, Ly108, PD-1 and Tim3 expression by NS3- and NS4-specific effector cells in the presence or absence of CD4 + T cell help. (C) Total hepatic cell numbers of all CD8+ T cells, NS3- and NS4-specific cells. (D) Expression of Eomes and T-bet and in general (all CD8), NS3- and NS4-specific CD8+ T cell subsets in CD40L blocked mice and controls at day 21 pi (n = 6). (E) LAG-3, Ly108, PD-1 and Tim3 expression by NS3- and NS4-specific effector cells in the presence or absence of CD40L interactions. (F) Expression of CD103 in general (all CD8), NS3- and NS4-specific CD8+ T cell subsets in CD4-depleted mice and controls at day 21 pi. Graphs show mean with SD. Statistics: unpaired two-tailed t test; * p<0.05, ** p<0.01, *** p<0.001, **** p<0.0001. (TIF) [file ppat.1012615.s005.tif]

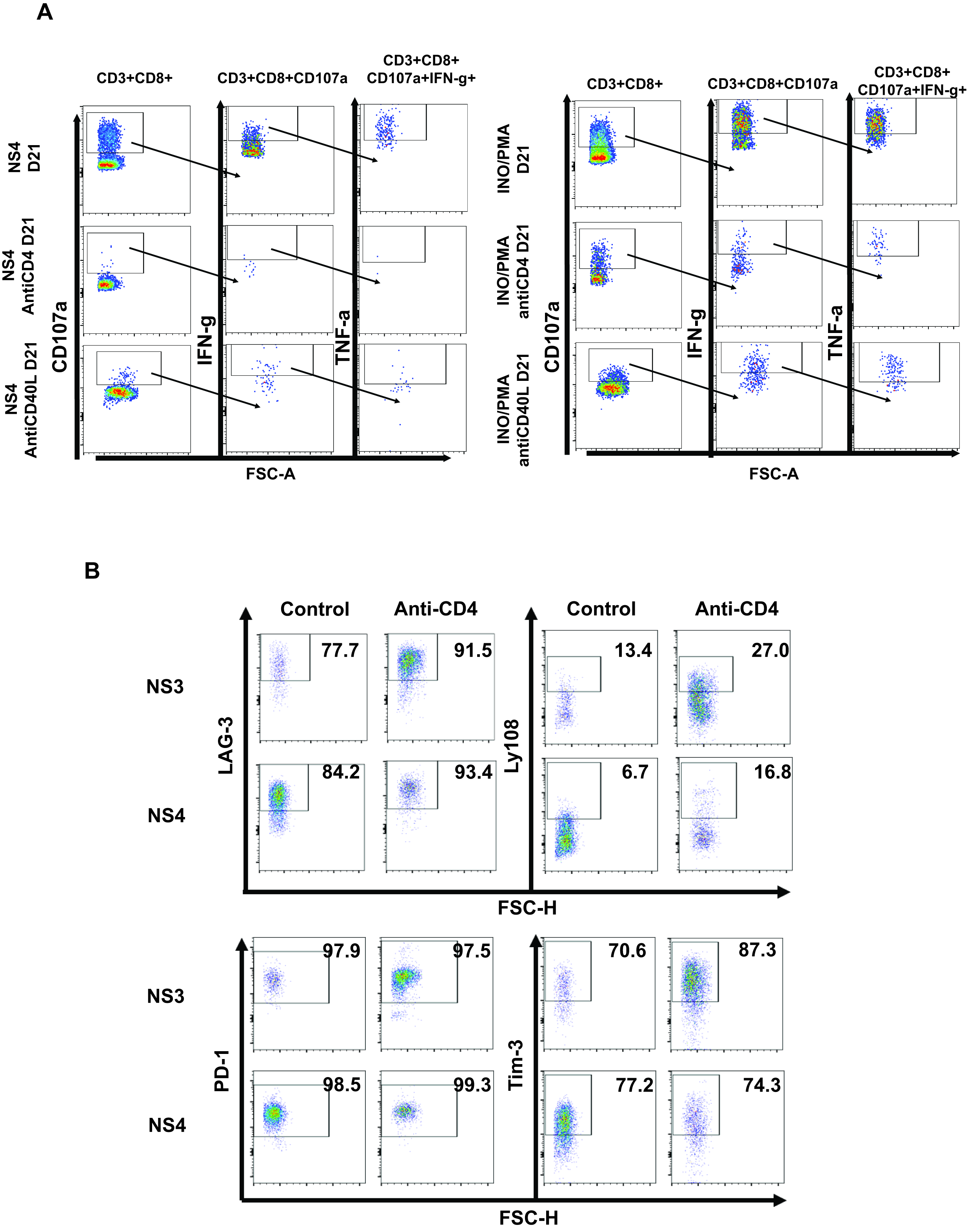

Supplement: S6 Fig — (A) Representative FACS plots showing polyfunctional (IFN-γ+TNF-α+CD107a+) effector functions after stimulation with the NS4 peptide or PMA/Ionomycin in CD8+ T cells from control mice, CD4-depleted mice and CD40L blocked mice. (B) Representative FACS plots (from concatenated samples from each group) showing LAG-3, PD-1, Ly108, TIM-3 expression on NS3- and NS4-specific CD8+ T cells from CD4-depleted mice and controls. (TIF) [file ppat.1012615.s006.tif]

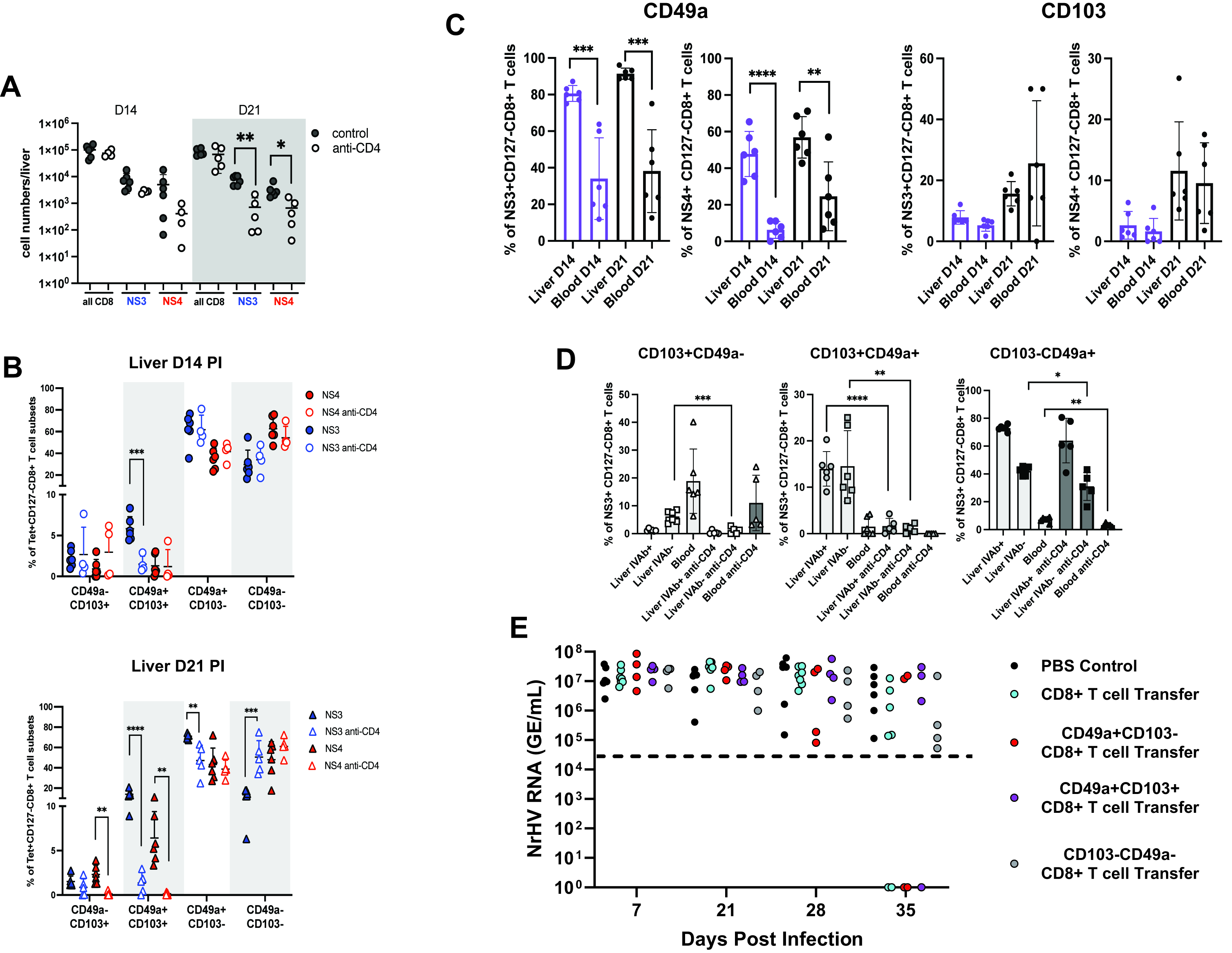

Supplement: S7 Fig — (A) Total hepatic cell numbers of all CD8+ T cells, NS3- and NS4-specific cells. (B) Frequencies of CD103/CD49a subsets within the NS3- and NS4-specific CD8+ T cell populations at day 14 and 21 pi in CD4-depleted mice and controls (n = 4–6). (C) CD49a and CD103 expression on liver and peripheral blood derived NS3- and NS4-specific effector cells at day 14 and 21 pi in WT control mice (n = 6). (D) Percentages of CD103/CD49a subsets within the NS3-specific populations from liver IVAb+, liver IVAb- and peripheral blood at 21 pi in CD4-depleted mice and controls (n = 6). (E) Adoptive transfer of sorted CD49a+CD103+, CD49a+CD103-, CD49a-CD103 populations (6x10^3^ cells/mouse, n = 4) or total CD8+ T cells (6.9x10^4^ cells/mouse, n = 7), or PBS (n = 6) into congenic CD8 knock-out (KO) mice at day 14 pi. Experimental outline similar to Fig 1D. Graphs show mean with SD or data points of individual mice. LOQ: limit of quantification. Statistics: unpaired two-tailed t test or one-way ANOVA with Tukey multiple comparison test; * p<0.05, ** p<0.01, *** p<0.001, **** p<0.0001. (TIF) [file ppat.1012615.s007.tif]

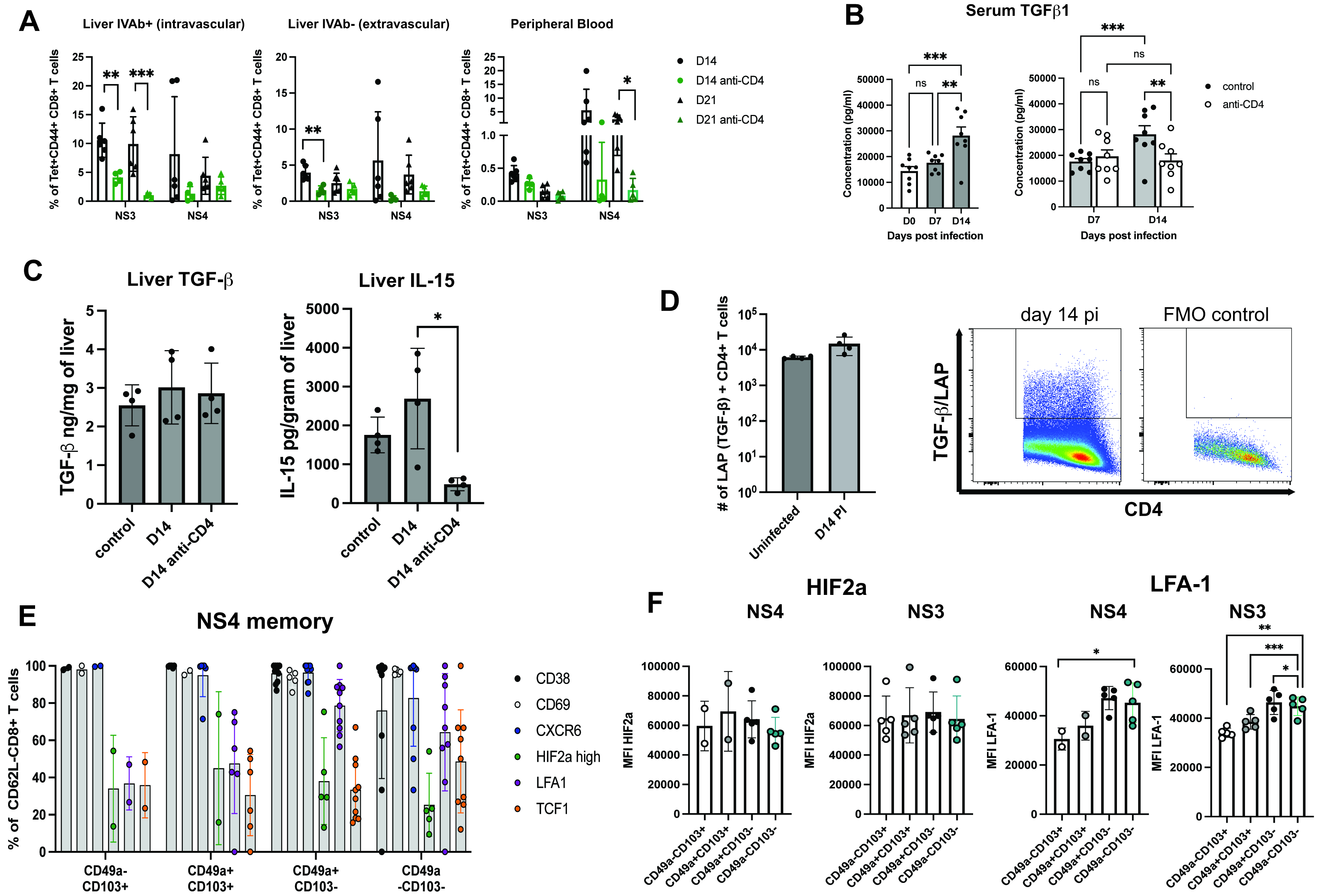

Supplement: S8 Fig — (A) Frequencies of NS3- and NS4-specific cells in the liver IVAb+, liver IVAb- compartment and peripheral blood at day 14 and 21 pi (n = 6). (B) Serum TGF-β levels at day 7 and day 14 pi (n = 8 per group). (C) Tissue TGF-β and IL-15 levels at day 14 pi (n = 4). (D) Flow cytometric analysis of TGF-β/LAP expression by hepatic CD4+ T cells at day 14 pi and in uninfected controls (n = 4). Left: total hepatic cell numbers. Right: Original FACS plot from day 14 pi as compared to FMO control. (E) CD38, CD69, CXCR6, LFA-1, HIF-2a and TCF-1 expression in the respective NS4+ memory CD103/CD49a subsets. (F) MFI (mean fluorescence intensity) of HIF-2a and LFA-1 in NS3+ and NS4+ memory CD103/CD49a subsets. Statistics: unpaired two-tailed t test or one-way ANOVA with Tukey multiple comparison test; * p< 0.05, ** p< 0.01, *** p<0.001, **** p<0.0001. (TIF) [file ppat.1012615.s008.tif]

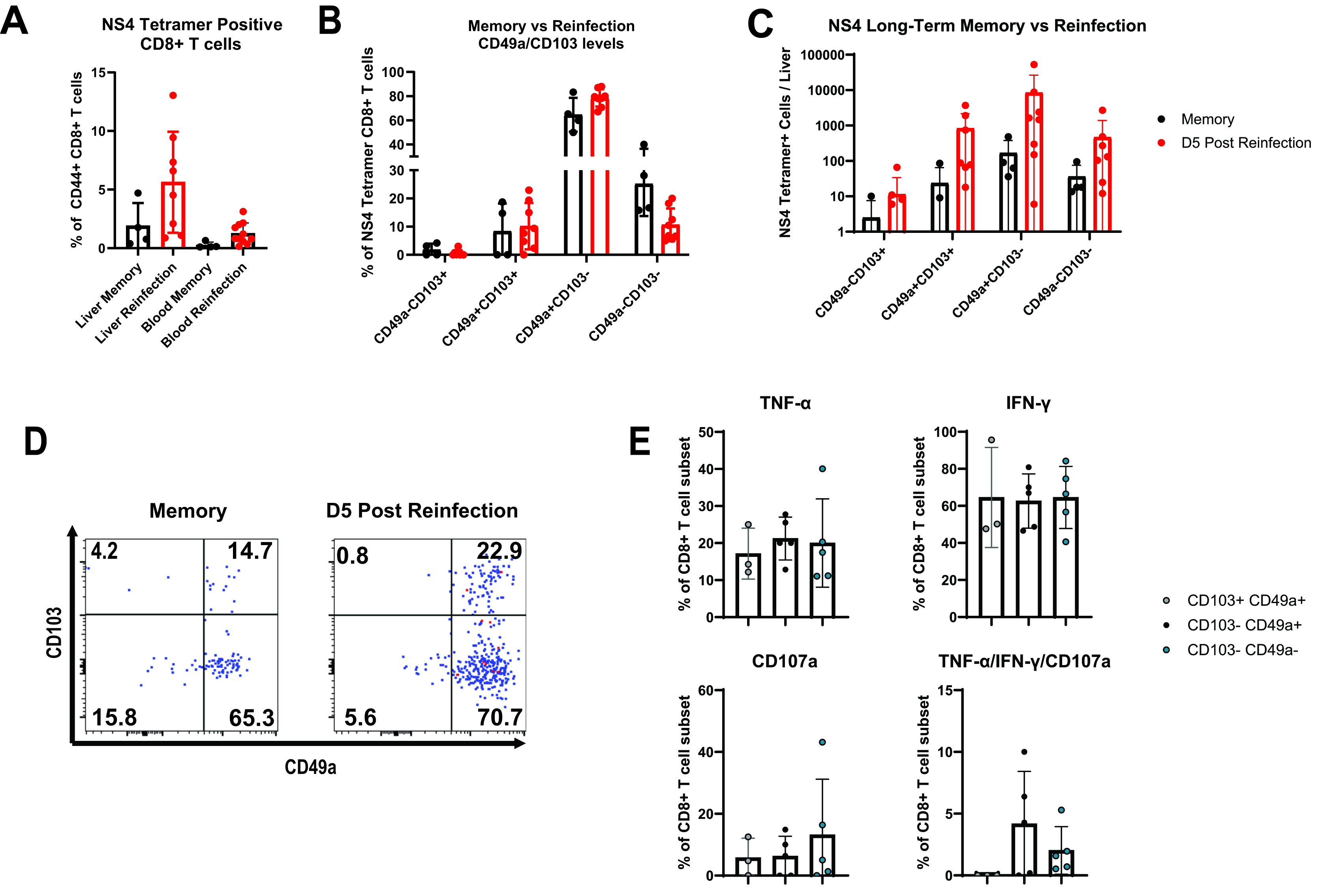

Supplement: S9 Fig — A) Percentages of NS4+ CD44+ CD8+ T cells in the liver and blood of memory mice or at 5 days post reinfection (n = 4–8). (B) Percentages of hepatic NS4+ CD49a/CD103 subsets from memory and reinfection mice(n = 4–8). (C) Total hepatic cell count of NS4+ CD49a/CD103 subsets. Graphs show mean with SD (n = 4–8). (D) FACS plots from representative samples of NS4+ CD49a/CD103 cells of memory and reinfection mice. (E) TNF-α, IFN-γ and/or CD107a expression of hepatic CD103/CD49a CD8+ T cell subsets after unspecific stimulation with PMA/Ionomycin at day 5 of secondary infection. (TIF) [file ppat.1012615.s009.tif]

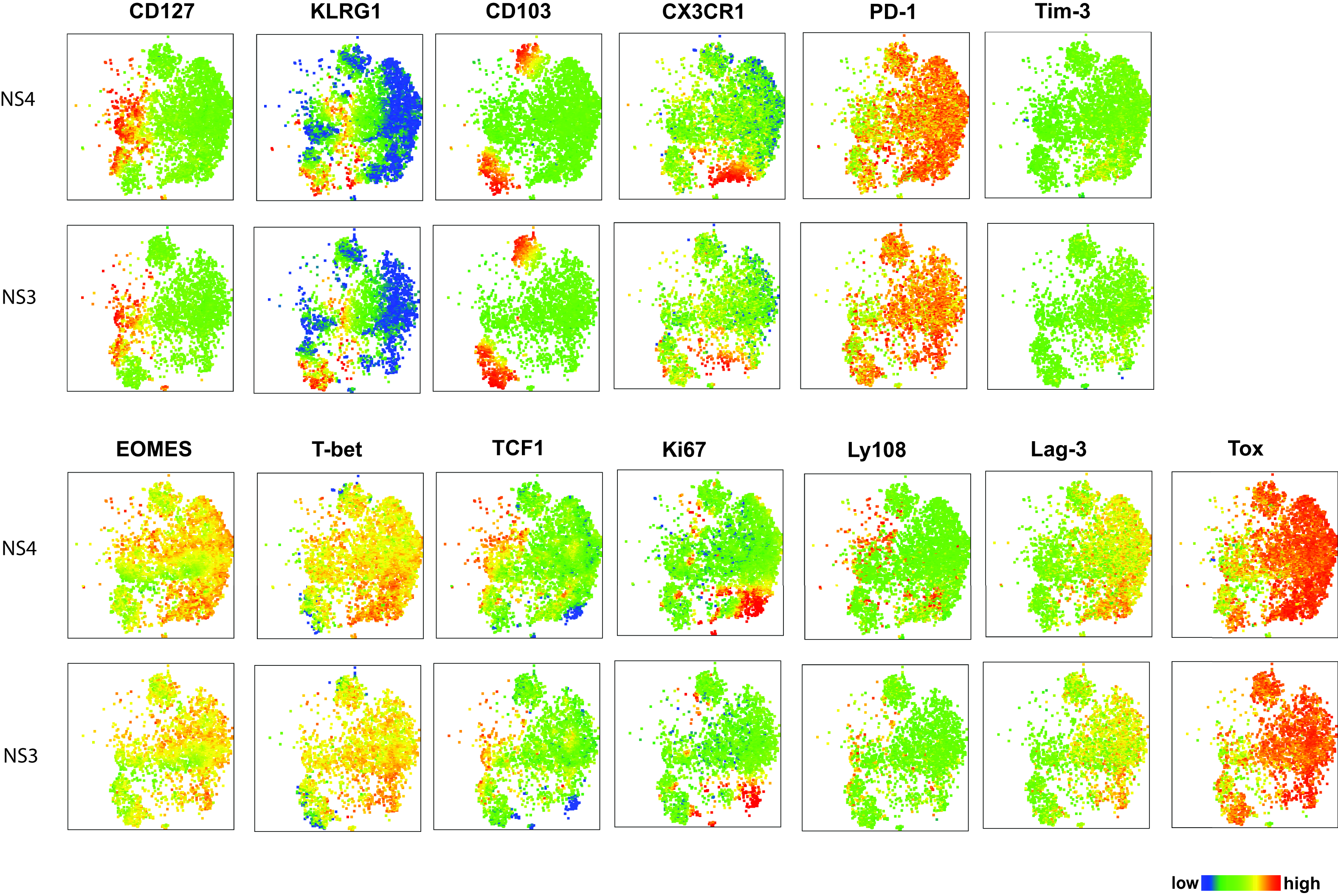

Supplement: S10 Fig — (TIF) [file ppat.1012615.s010.tif]

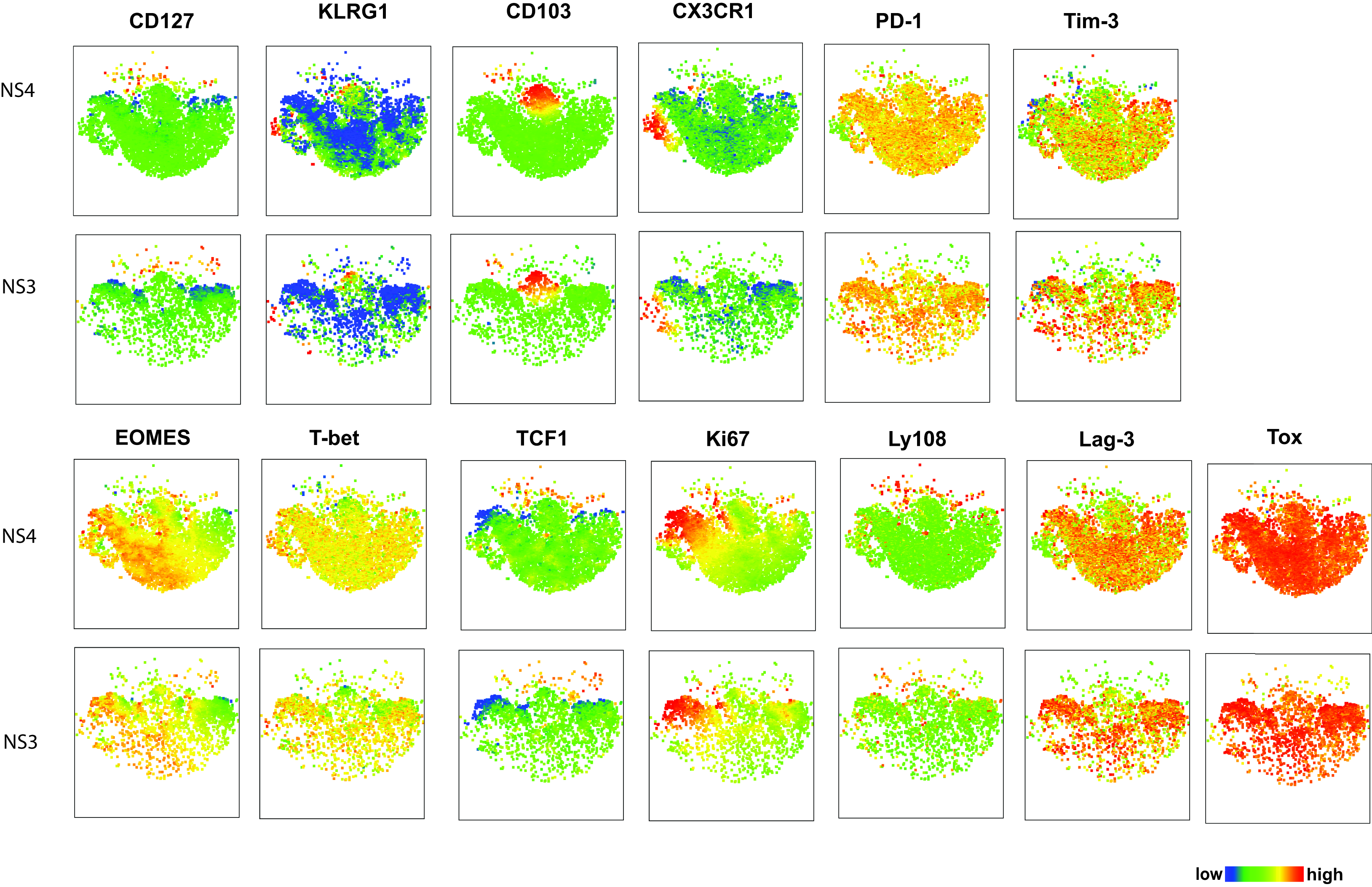

Supplement: S11 Fig — (TIF) [file ppat.1012615.s011.tif]
